# Supplementary material for: Evidence for opposing selective forces operating on human-specific duplicated TCAF genes in Neanderthals and humans
Source: Nat Commun. 2021 Aug 25;12:5118. doi: 10.1038/s41467-021-25435-4 (PMC8387397; doi:10.1038/s41467-021-25435-4)
Supplement: Supplementary file 1 — Supplementary Information [file 41467_2021_25435_MOESM1_ESM.pdf]

Supplementary Information

Evidence for opposing selective forces operating on human-specific duplicated *TCAF* genes in Neanderthals and humans

PingHsun Hsieh,[1*] Vy Dang,[1,†] Mitchell R. Vollger,[1] Yafei Mao,[1] Tzu-Hsueh Huang,[1] Philip C. Dishuck,[1] Carl Baker[1], Stuart Cantsilieris,[1,‡] Alexandra P. Lewis,[1] Katherine M. Munson, [1] Melanie Sorensen,[1] AnneMarie E. Welch,[1,ǂ] Jason G. Underwood,[1,2] Evan E. Eichler[1,3*]

*Co-corresponding authors

Correspondence: hsiehph@uw.edu

eee@gs.washington.edu

Affiliations:

1. Department of Genome Sciences, University of Washington School of Medicine, Seattle, WA, USA

2. Pacific Biosciences (PacBio) of California, Incorporated, Menlo Park, CA, USA

3. Howard Hughes Medical Institute, University of Washington, Seattle, WA, USA

† Current address: Institute for Cell and Molecular Biology, University of Texas, Austin, TX, USA

‡ Current address: Centre for Eye Research Australia, Department of Surgery (Ophthalmology), University of Melbourne, Royal Victorian Eye and Ear Hospital, East Melbourne, VIC, Australia

ǂ Current address: Brain and Mitochondrial Research, Murdoch Children's Research Institute, Royal Children's Hospital, Melbourne, VIC, Australia.

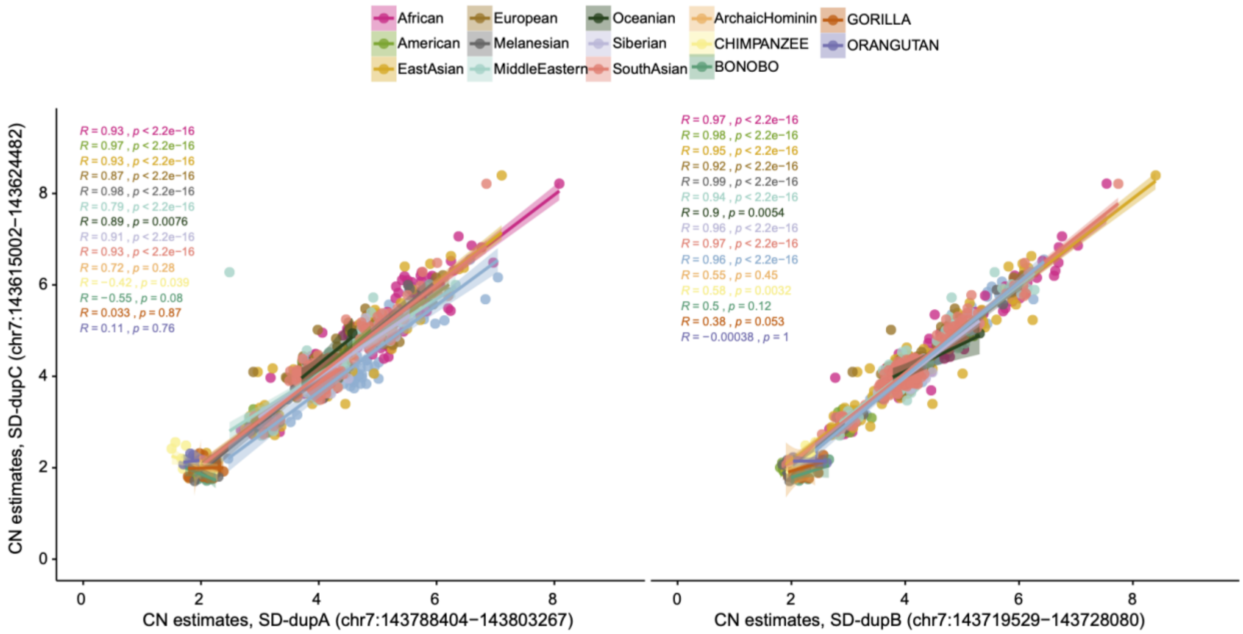

Supplementary Fig. 1. Pairwise joint distributions among copy number (CN) estimates of SD-DupA, SD-DupB, and SD-DupC variants for all human and nonhuman samples. Each symbol is the data from an individual. Linear regression lines and their 95% C.I. region were drawn for individual populations. Pearson's correlation coefficients (R) and unadjusted p values (two-tailed) were computed for individual population/species, including African (N=192 samples), Native American (N=75), East Asian (N=223), European (N=180), Melanesian (N=29), Middle Easterner (N=146), Oceanian (N=7), Siberian (N=45), South Asian (N=205), archaic hominin (N=4), chimpanzee (N=24), Bonobo (N=11), Gorilla (N=26), and Orangutan (N=10) individuals. For a population/species, the shaded area represents the 95% confidence interval of the fitted regression line.

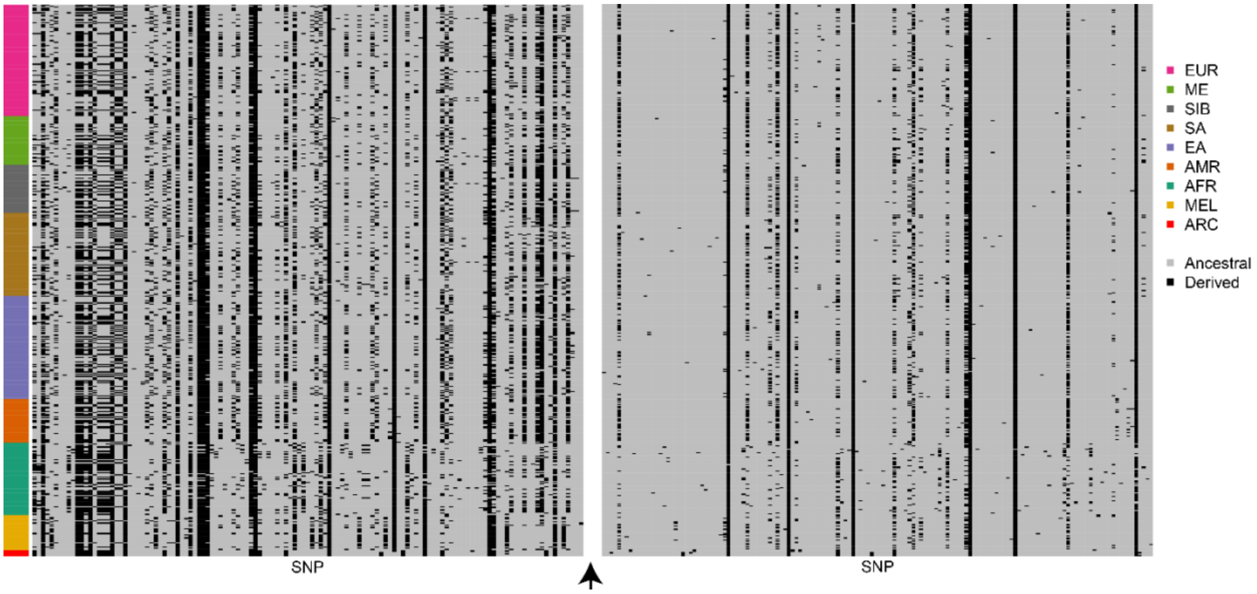

Supplementary Fig. 2. No evidence showing archaic introgression at the *TCAF* locus. Haplotypes from both archaic (bottom rows indicated by the red bars on the left) and modern human samples were constructed using single-nucleotide variants (SNVs) from the 20 kbp unique diploid sequences flanking the two sides of the *TCAF* SD regions (black arrow; chr7:143521769- 143874696, GRCh38) and phased computationally using BEAGLE (v5.1). Population IDs: EUR (European), ME (Middle Eastern), SIB (Siberian), SA (South Asian), EA (East Asian), AMR (American), AFR (African), MEL (Melanesian), ARC (archaic hominin).

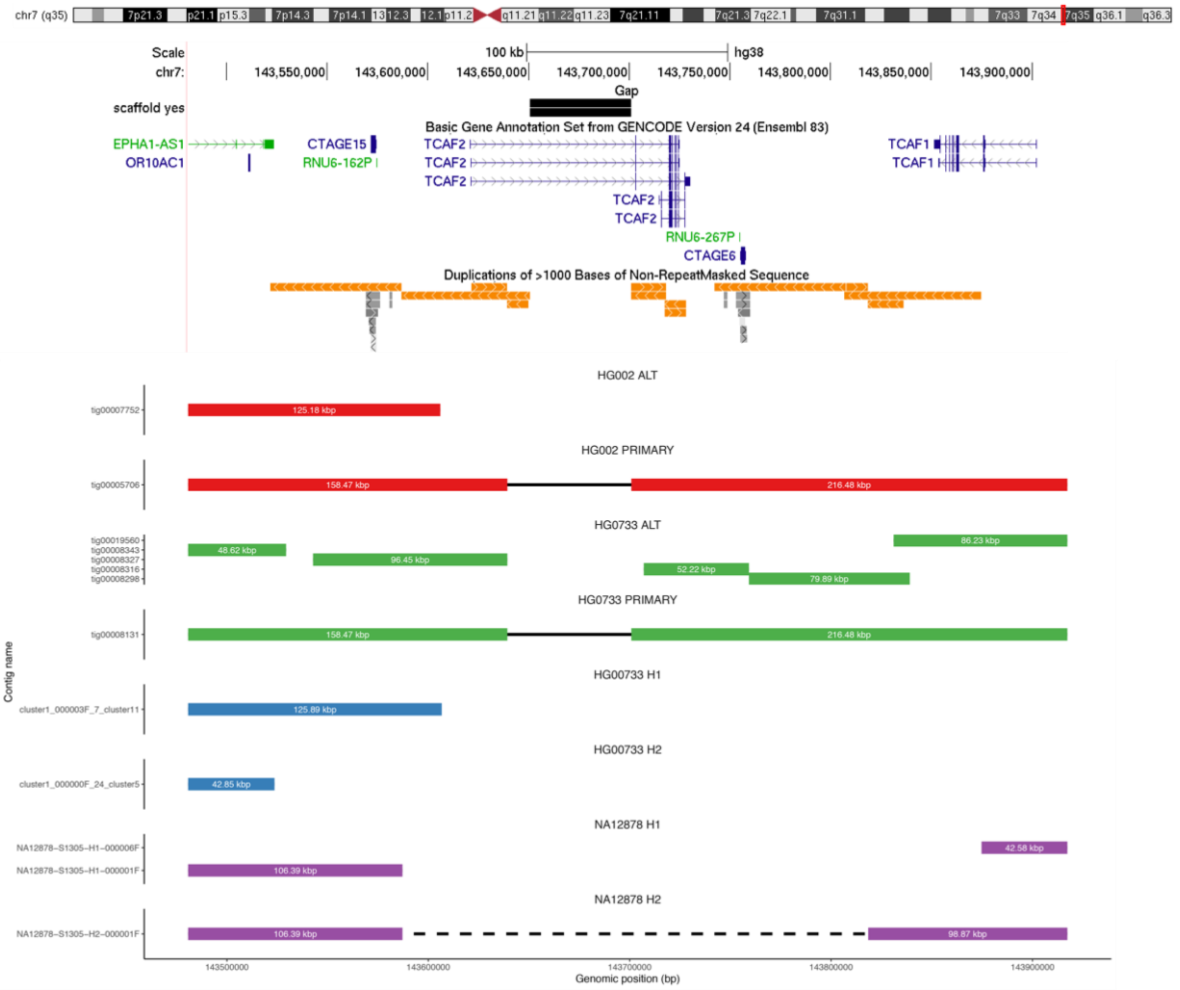

Supplementary Fig. 3. Difficulty of assembling *TCAF* SD haplotypes using publically available PacBio high-fidelity (HiFi) long-read data and out-of-box assembly pipelines. Contigs from four recently published long-read assemblies (Supplementary Data 2) were mapped to the human reference genome GRCh38 using minimap2 (v2.17-r941). In three of the four assemblies, there is one contig in each case mapped across the *TCAF* region of GRCh38, but all four diploid assemblies fail to create haplotype-resolved, contiguous sequences. Black horizontal solid lines indicate missing sequences in the GRCh38, while the black dashed line indicates a putative deletion event with respect to the GRCh38.

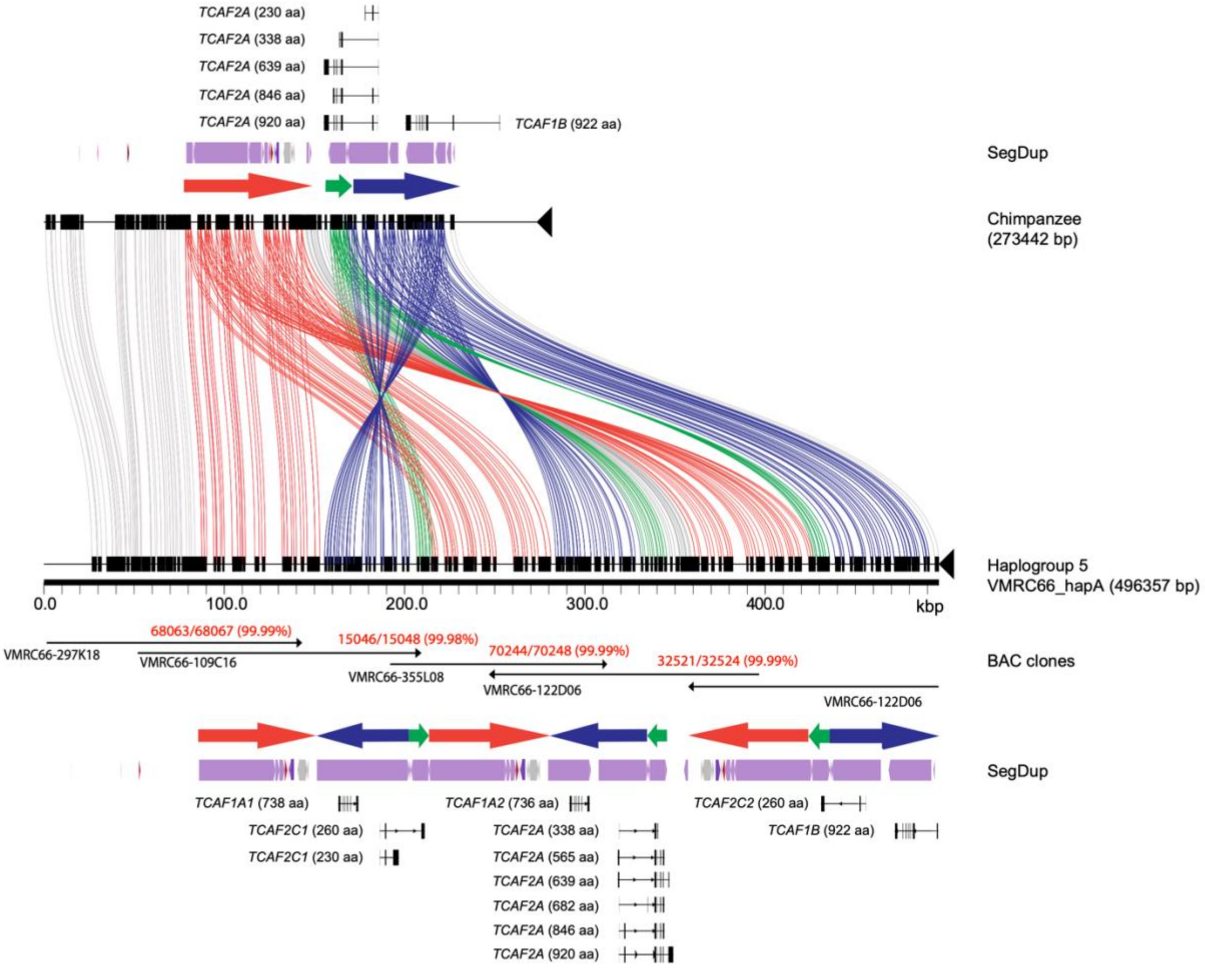

Supplementary Fig. 4. Miropeats analysis reveals structure similarity and dissimilarity at the 7q35 *TCAF* locus between Haplogroup 5 and the chimpanzee haplotype. Colored arrows are annotated *TCAF* SDs and lines connecting the sequences show regions of homology. Additional annotations include segmental duplication (SegDup) tracks and the predicted gene models using FLNC transcripts from the chimpanzee lymphoblast cells and six human tissues for the chimpanzee and Haplogroup 5 sequences, respectively (Methods). Note that the numbers above overlapping between two BAC clones indicate the percent sequence identity (#identical bases/total bases).

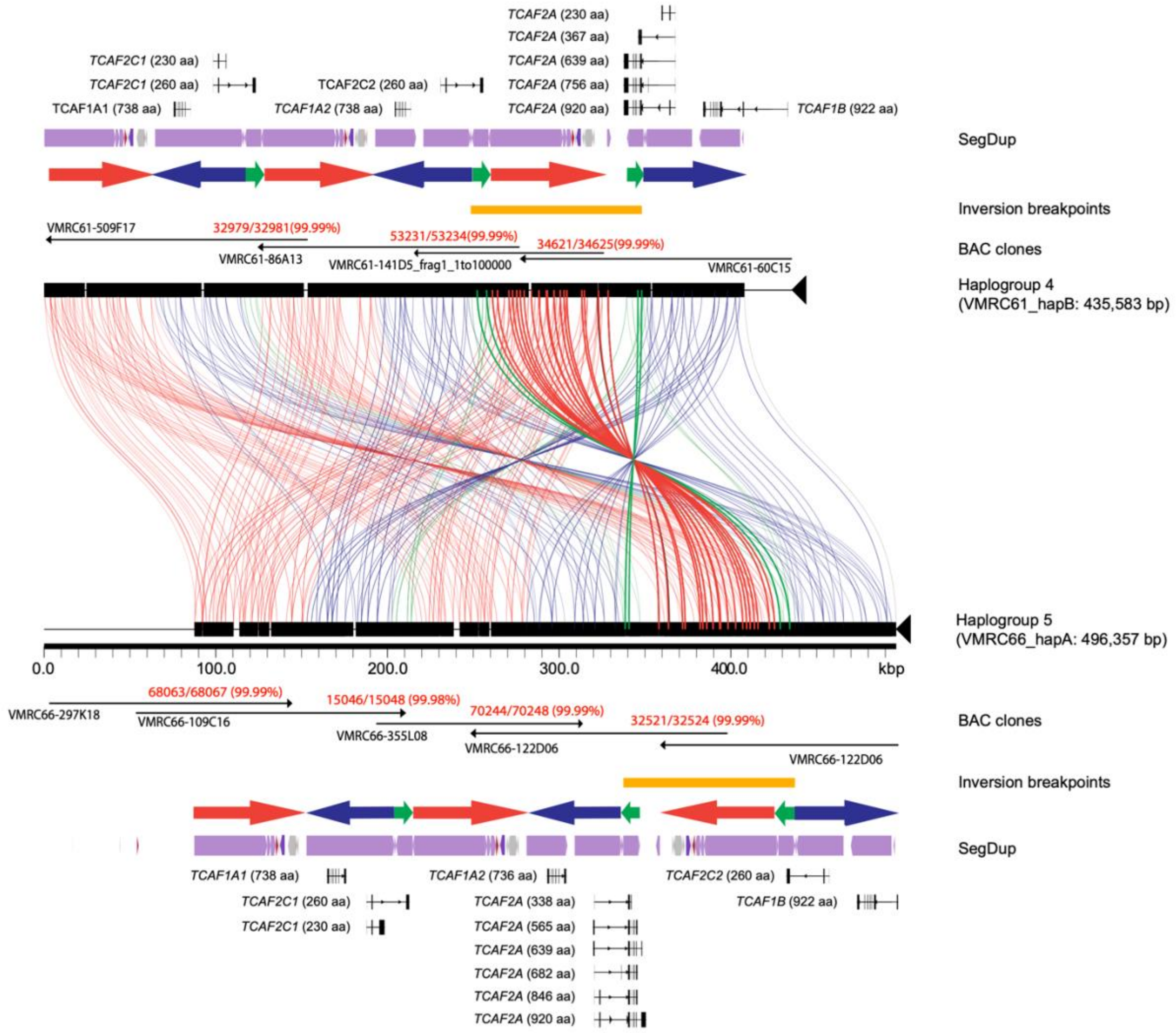

Supplementary Fig. 5. Miropeats analysis reveals structure similarity and dissimilarity at the 7q35 *TCAF* locus between Haplogroups 4 and 5. Colored arrows are annotated *TCAF* SDs and lines connecting the sequences show regions of homology. The orange bar indicates the location of a 100 kbp inversion between these two haplogroups. Additional annotations include segmental duplication (SegDup) tracks and the predicted gene models using FLNC transcripts from six human tissues for both haplotypes (Methods). Note that the numbers above overlapping between two BAC clones indicate the percent sequence identity (#identical bases/total bases).

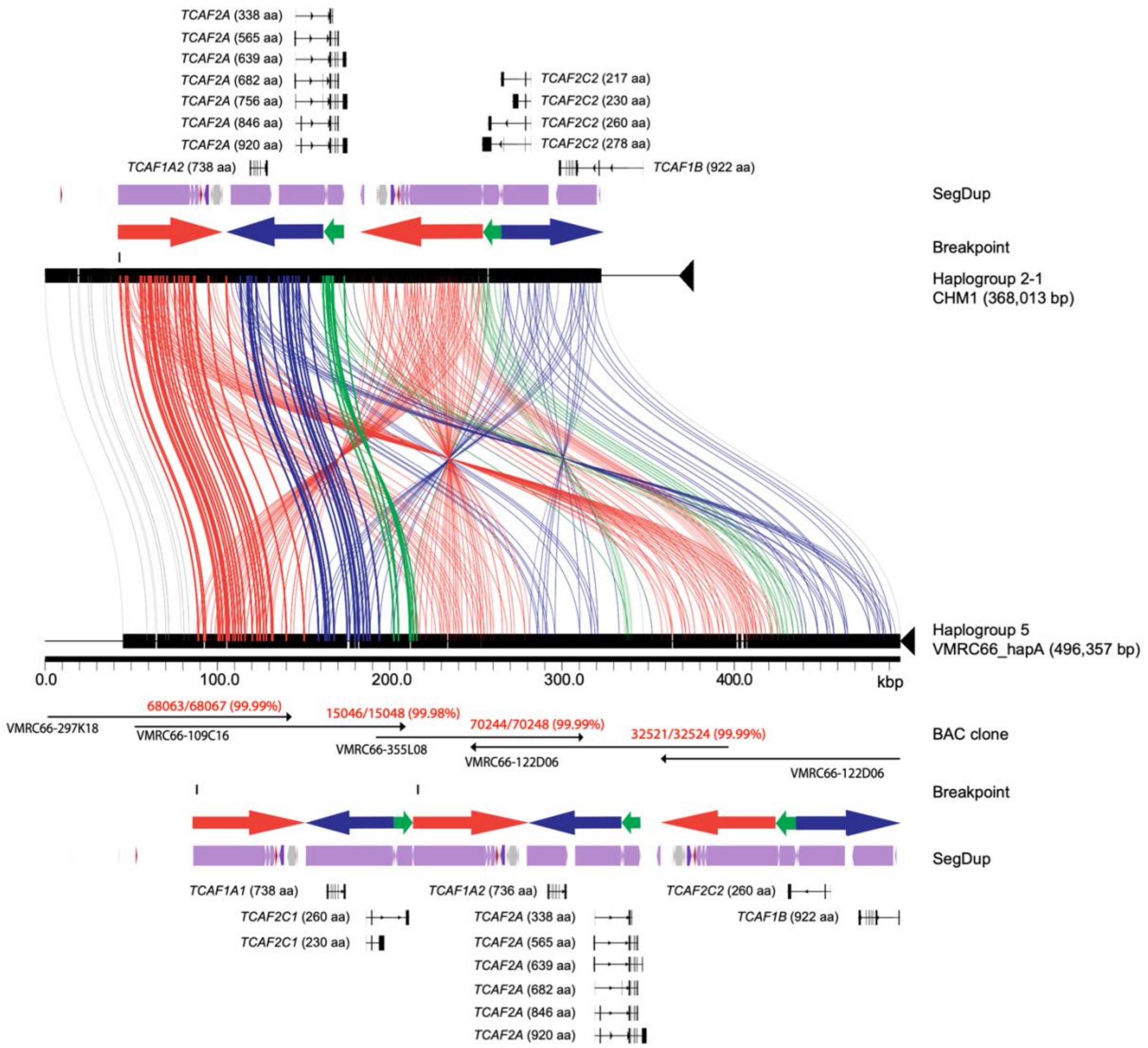

Supplementary Fig. 6. Miropeats analysis reveals structure similarity and dissimilarity at the 7q35 *TCAF* locus between Haplogroups 2-1 and 5. Colored arrows are annotated *TCAF* SDs and lines connecting the sequences show regions of homology. The vertical black bars indicate the putative breakpoints for a 129 kbp deletion event in Haplogroup 2-1 with respect to Haplogroup 5. Additional annotations include SegDup tracks and the predicted gene models using FLNC transcripts from six human tissues for both haplotypes (Methods). Note that the numbers above overlapping between two BAC clones indicate the percent sequence identity (#identical bases/total bases).

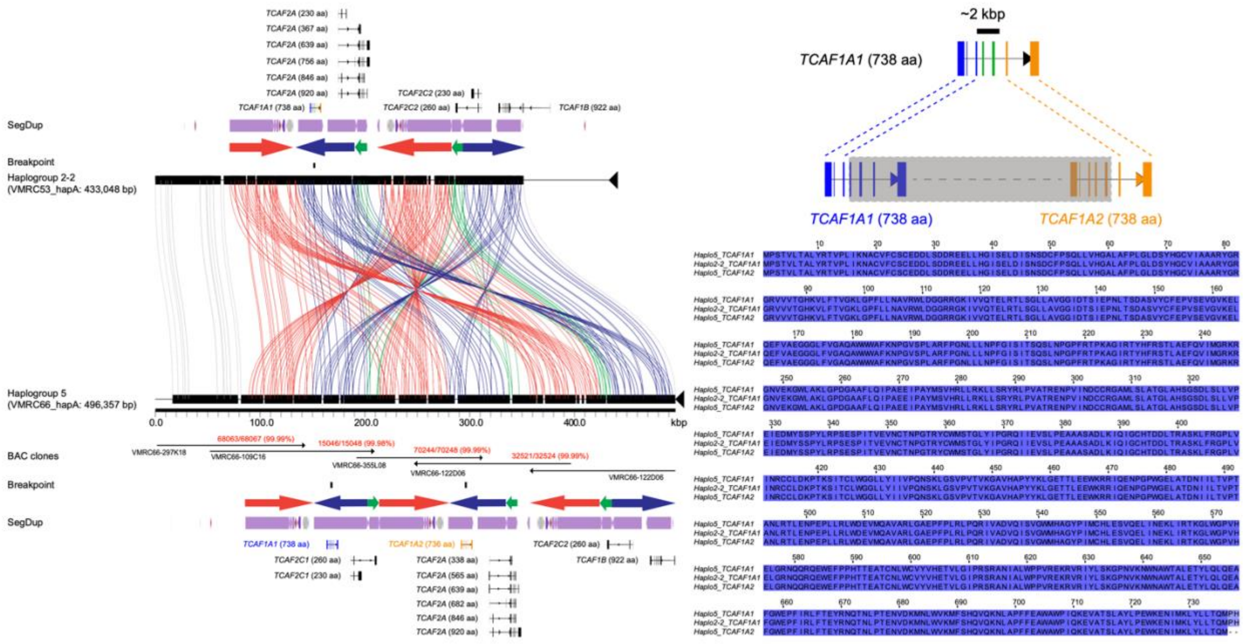

Supplementary Fig. 7. Miropeats analysis reveals structure similarity and dissimilarity at the 7q35 *TCAF* locus between Haplogroups 2-2 and 5. Colored arrows are annotated *TCAF* SDs and lines connecting the sequences show regions of homology. The vertical black bars indicate the putative breakpoints for a >130 kbp deletion event in Haplogroup 2-2 with respect to Haplogroup 5. The right panel shows that the *TCAF1A1* copy in Haplogroup 2-2 is a fusion of the *TCAF1A1* and *TCAF1A2* copies in Haplogroup5. Additional annotations include SegDup tracks and the predicted gene models using FLNC transcripts from six human tissues for both haplotypes (Methods). Note that the numbers above overlapping between two BAC clones indicate the percent sequence identity (#identical bases/total bases).

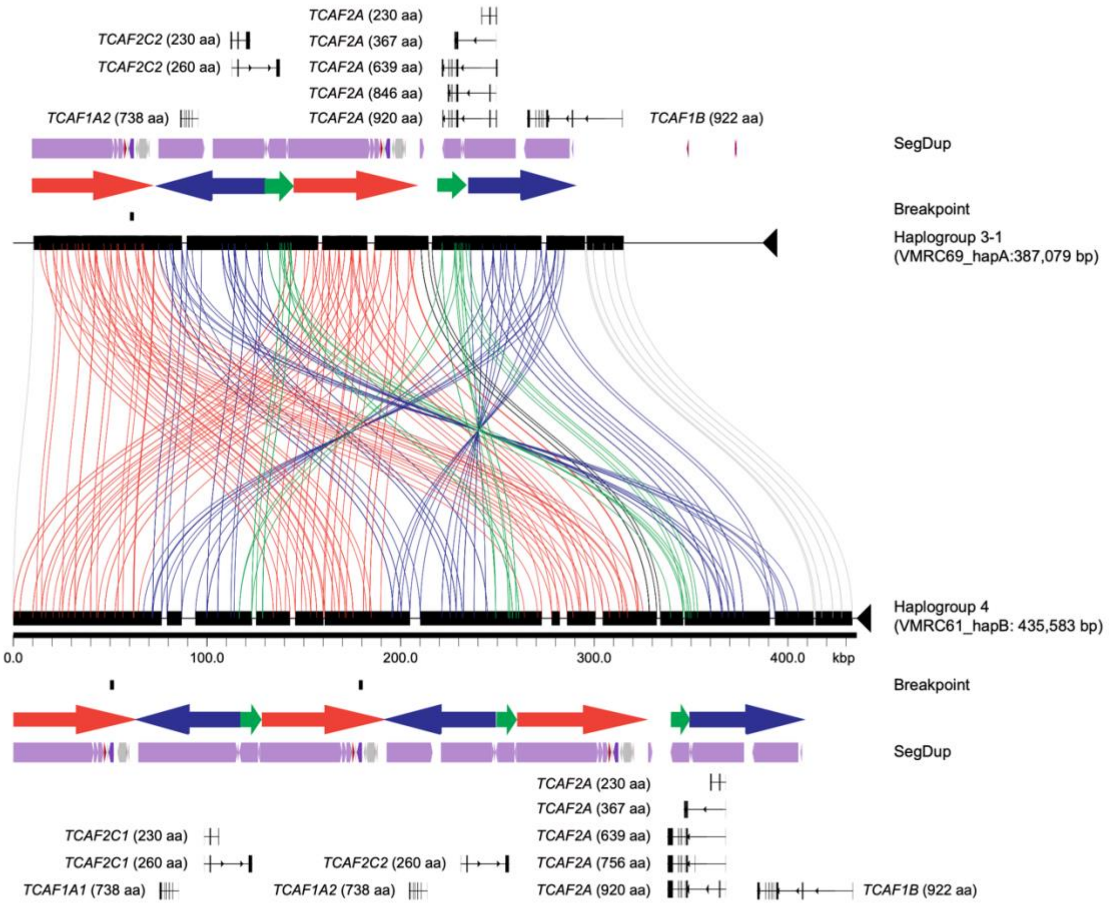

Supplementary Fig. 8. Miropeats analysis reveals structure similarity and dissimilarity at the 7q35 *TCAF* locus between Haplogroups 3-1 and 4. Colored arrows are annotated *TCAF* SDs and lines connecting the sequences show regions of homology. The vertical black bars indicate the putative breakpoints for a ~131 kbp deletion event in Haplogroup 3-1 with respect to Haplogroup 4. Note that the two inferred breakpoints on Haplogroup 4 intersect with two identical (100%) *CTAGE* sequences. Additional annotations include SegDup tracks and the predicted gene models using FLNC transcripts from six human tissues for both haplotypes (Methods). Note that the numbers above overlapping between two BAC clones indicate the percent sequence identity (#identical bases/total bases).

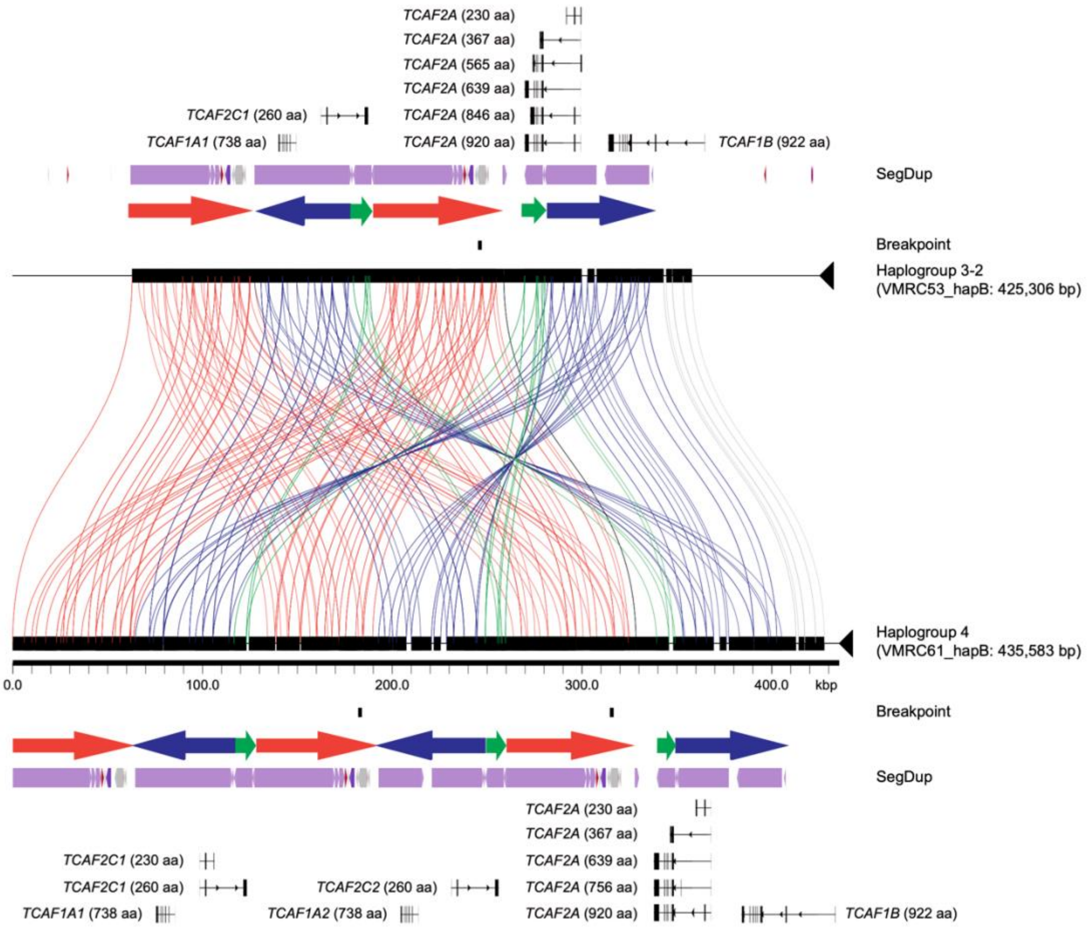

Supplementary Fig. 9. Miropeats analysis reveals structure similarity and dissimilarity at the 7q35 *TCAF* locus between Haplogroups 3-2 and 4. Colored arrows are annotated *TCAF* SDs and lines connecting the sequences show regions of homology. The vertical black bars indicate the putative breakpoints for a >134 kbp deletion event in Haplogroup 3-2 with respect to Haplogroup 4. Additional annotations include SegDup tracks and the predicted gene models using FLNC transcripts from six human tissues for both haplotypes (Methods). Note that the numbers above overlapping between two BAC clones indicate the percent sequence identity (#identical bases/total bases).

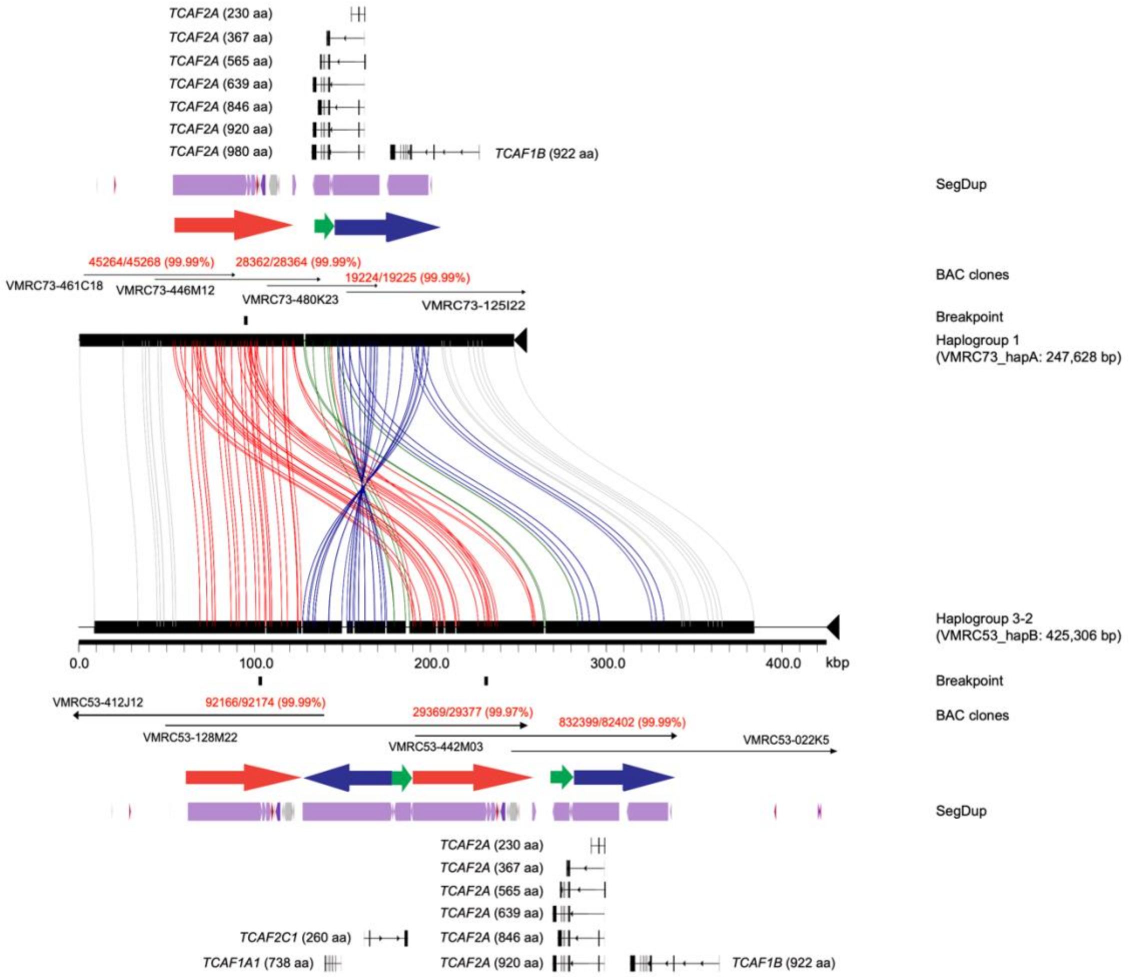

Supplementary Fig. 10. Miropeats analysis reveals structure similarity and dissimilarity at the 7q35 *TCAF* locus between Haplogroups 1 and 3-2. Colored arrows are annotated *TCAF* SDs and lines connecting the sequences show regions of homology. The vertical black bars indicate the putative breakpoints for a ~130 kbp deletion event in Haplogroup 1 with respect to Haplogroup 3-2. Additional annotations include SegDup tracks and the predicted gene models using FLNC transcripts from six human tissues for both haplotypes (Methods). Note that the numbers above overlapping between two BAC clones indicate the percent sequence identity (#identical bases/total bases).

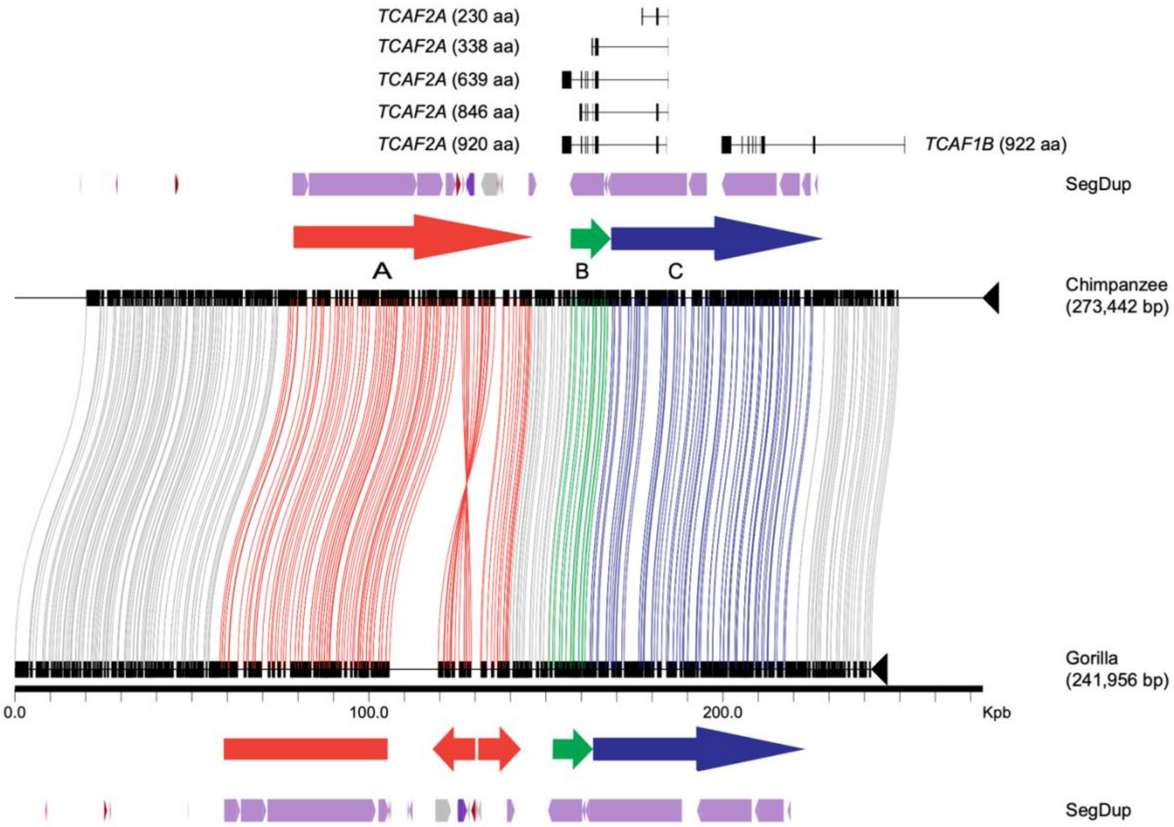

Supplementary Fig. 11. Miropeats analysis reveals structure similarity and dissimilarity at the 7q35 *TCAF* locus between the chimpanzee and gorilla haplotypes. Colored arrows are annotated *TCAF* SDs and lines connecting the sequences show regions of homology. Additional annotations include SegDup tracks and the predicted gene models using FLNC transcripts from the chimpanzee lymphoblast cells for the chimpanzee haplotype (Methods).

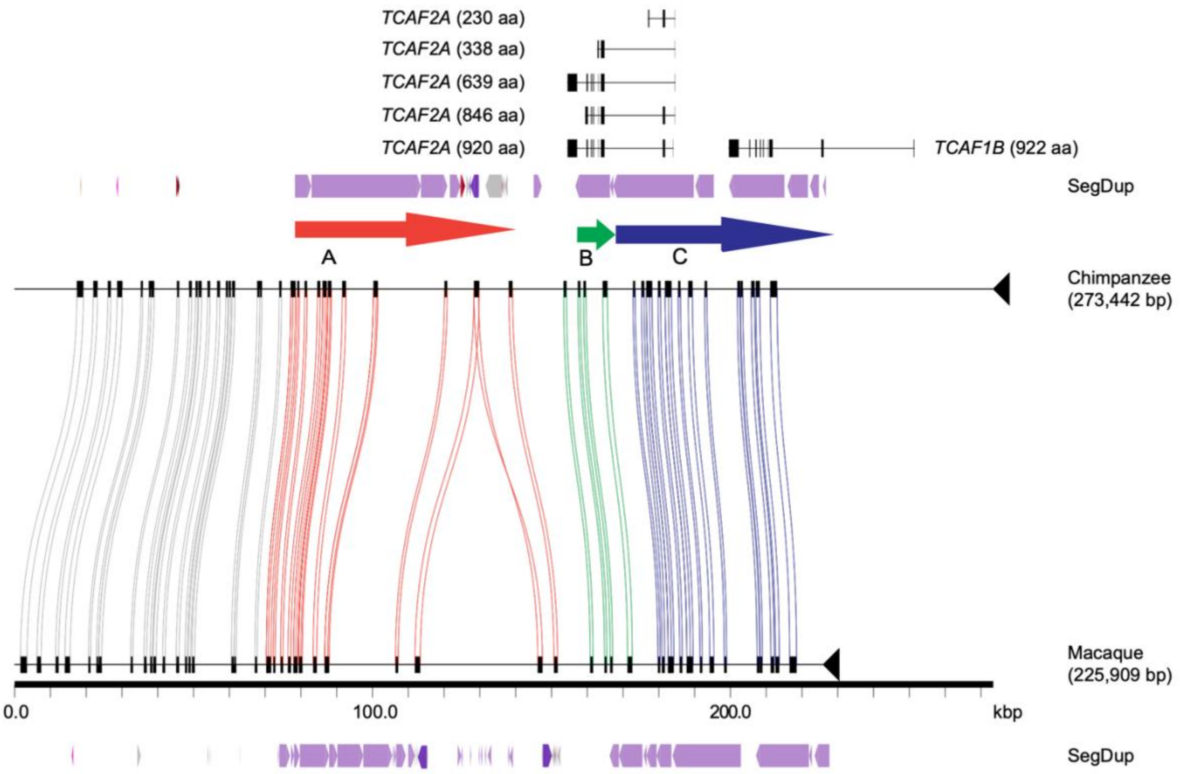

Supplementary Fig. 12. Miropeats analysis reveals structure similarity and dissimilarity at the 7q35 *TCAF* locus between the chimpanzee and macaque haplotypes. Colored arrows are annotated *TCAF* SDs and lines connecting the sequences show regions of homology. Additional annotations include SegDup tracks and the predicted gene models using FLNC transcripts from the chimpanzee lymphoblast cells for the chimpanzee haplotype (Methods).

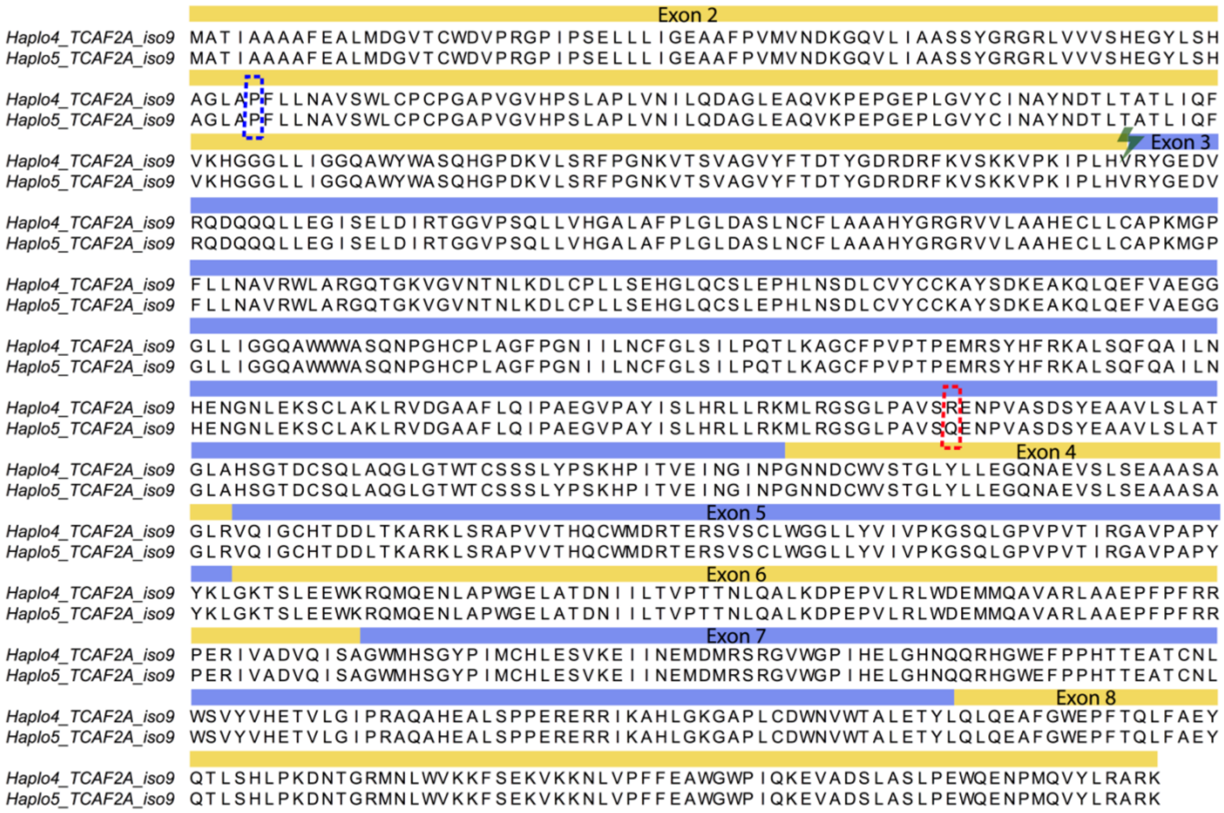

Supplementary Fig. 13. Sequence alignment between Haplogroups 4 and 5 TCAF2A amino acid sequences. Only two variants were found between the coding sequences. The blue dashed box indicates the synonymous difference at exon 2, while the red dashed box shows the nonsynonymous change at exon 3, which is beyond the putative inversion breakpoints (Supplementary Fig. 6). The green lightning bolt represents the inversion breakpoints at the 3rd intron between the 2nd and 3rd exons.

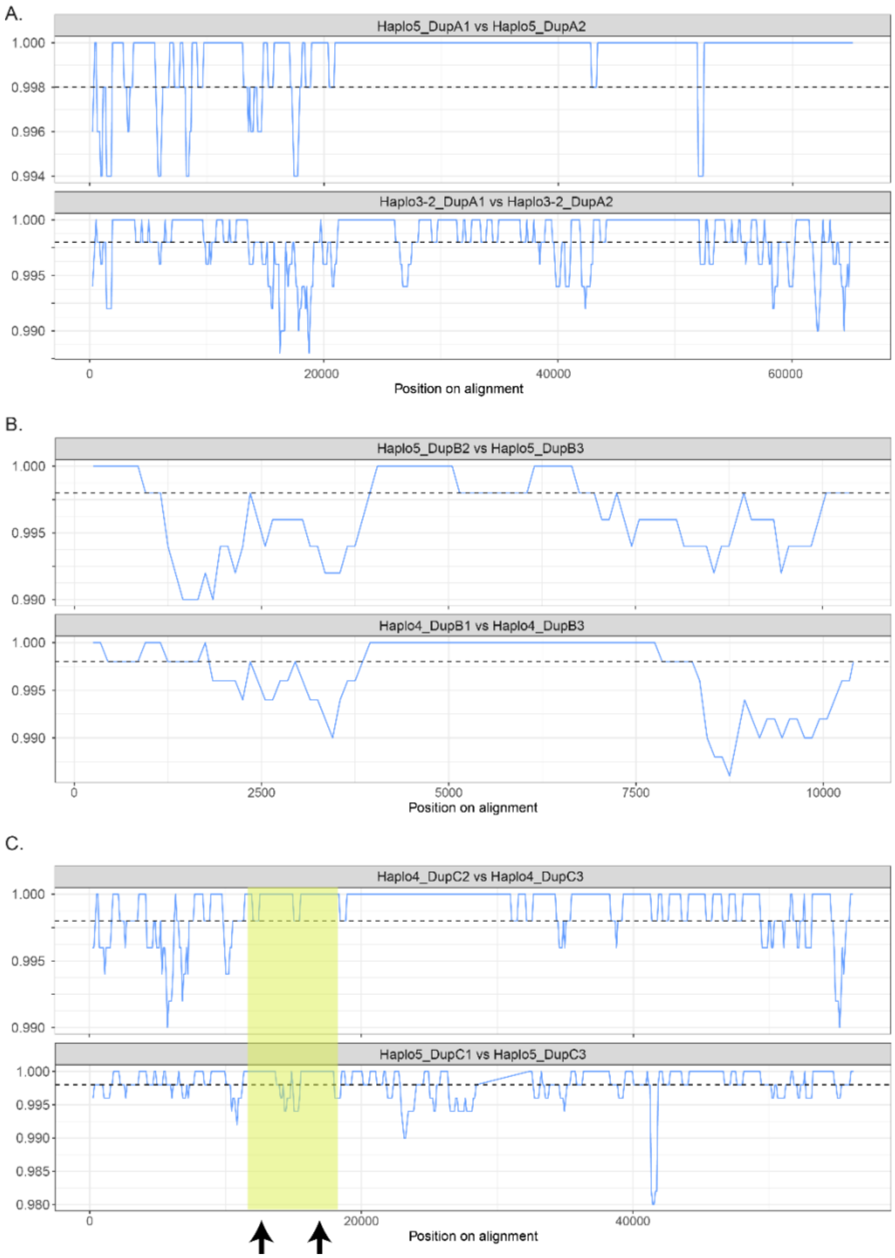

Supplementary Fig. 14. Evidence of interlocus gene conversion (IGC) between *TCAF* SD paralogs. Each panel shows pairwise sequence identities (blue curves) over 500 bp windows (sliding by 100 bp) between individual *TCAF* SDs from BAC haplotypes. Dashed lines indicate 99.8% sequence identity. Sudden increases to 100% sequence identity between pairs of *TCAF* SDs were used to support the observations of IGC incidents. (A) *TCAF* DupA segments. (B) *TCAF* DupB segments. (C) *TCAF* DupC segments. The light green rectangle highlights a putative IGC segment corresponding to the latitude-correlated IGC locus described in the main text and Supplementary Figs. 15-17.

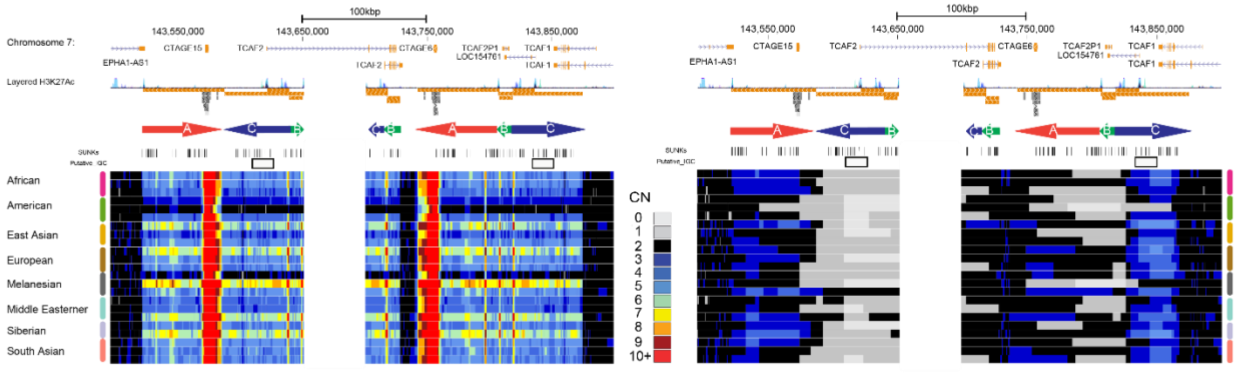

Supplementary Fig. 15. Heatmaps for overall and paralog-specific CN trajectories across human populations. CN estimated using read-depth-based genotyping method. Each row of the CN heatmaps represents the CN of a sample over the *TCAF* locus (left: overall CN; right: paralog-specific CN, which is estimated using singly unique nucleotide k-mers [SUNKs]). The colored arrows (A, B, and C) represent the three major *TCAF* SD blocks in this region. The white area in the middle represents the gap present in the human reference genome (GRCh38). The two white boxes in both panels show the putative IGC event that correlates with latitudinal locations of global populations. The left white box shows the acceptor site (chr7:143,615,002−143,624,482), while the right one indicates the donor locus (chr7:143,833,163−143,842,658). Note that the gap present in the reference distorts the CN trajectories around it in the heatmap.

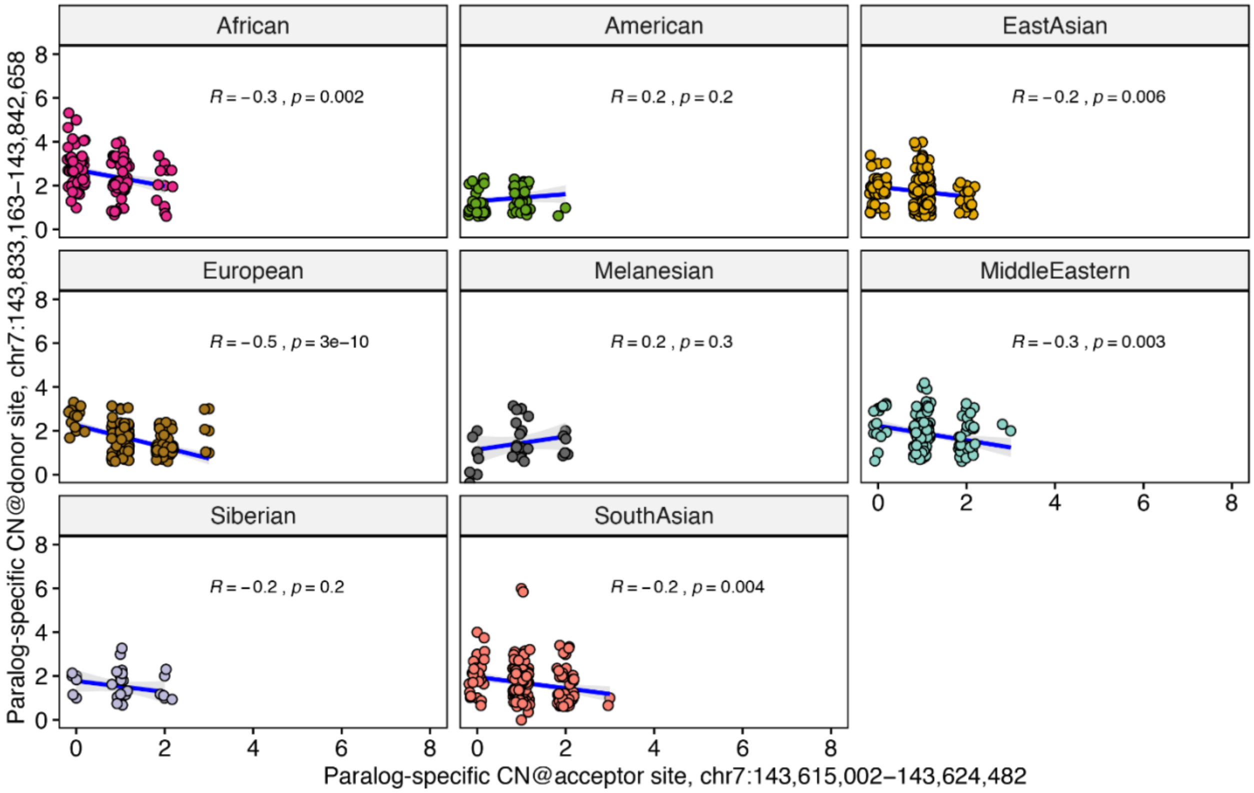

Supplementary Fig. 16. Significant negative correlations in paralog-specific CN estimates between the putative IGC donor and acceptor sites across multiple continental populations. Each dot represents the paralog-specific CN estimates of the donor (y-axis) and acceptor (x-axis) sites for a given sample using a read-depth-based approach and the information from SUNKs. Correlation coefficients and unadjusted p values (two-tailed) were computed using Person's correlation test for African (N=192 samples), Native American (N=75), East Asian (N=223), European (N=180), Melanesian (N=29), Middle Easterner (N=146), Siberian (N=45), and South Asian (N=205) individuals. In each panel, the shaded area represents the 95% confidence interval of the fitted regression line.

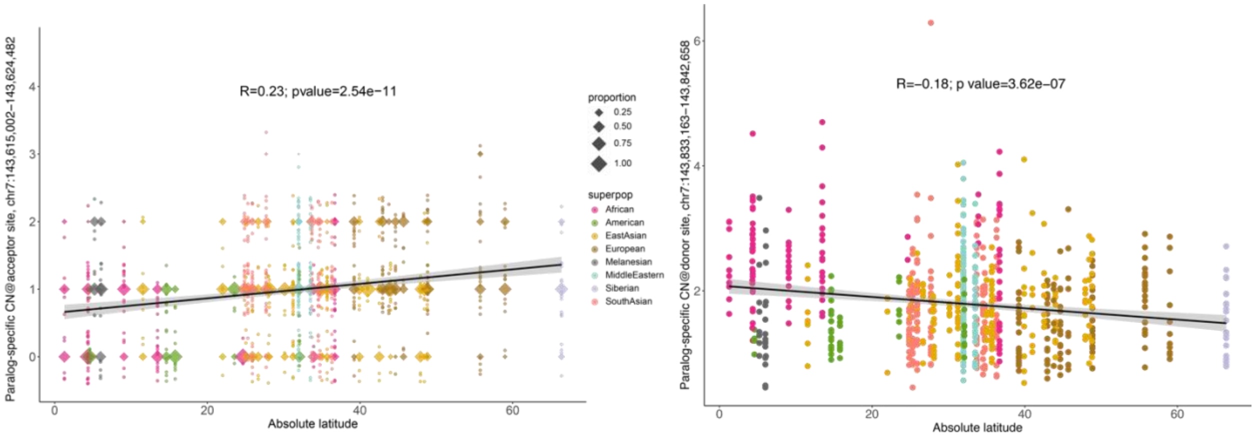

Supplementary Fig. 17. Significant correlation between paralog-specific CN at the putative IGC loci and latitudinal locations of human populations. The left panel shows a significant positive correlation between the paralog-specific CN estimates at the IGC acceptor site and latitudinal locations of 54 human populations, while the opposite trend is observed for the IGC donor site (the right panel). Pearson's correlation coefficients (R) and unadjusted p values (two-tailed) were computed across African (N=192 samples), Native American (N=75), East Asian (N=223), European (N=180), Melanesian (N=29), Middle Easterner (N=146), Siberian (N=45), and South Asian (N=205) individuals. The latitudes of these 54 populations can be found in Supplementary Data 8. Shaded gray areas represent the 95% confidence intervals of the fitted regression lines.

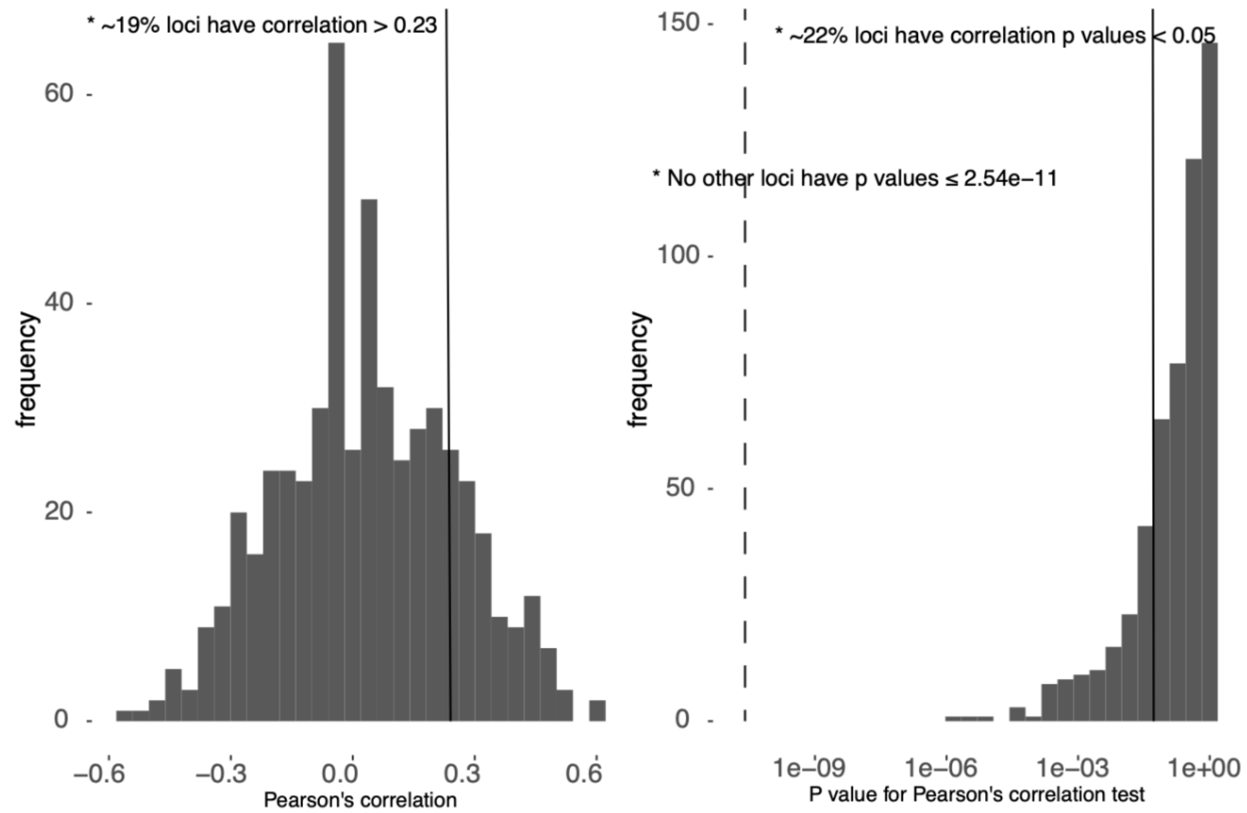

Supplementary Fig. 18. Tests of correlation between CN estimates and latitudinal locations of populations for 1,000 random SD loci. Left panel: the distribution of Pearson's correlation coefficients for the randomly drawn loci; the vertical line indicates the observed Pearson's correlation coefficient of 0.23 for the putative IGC acceptor locus (Supplementary Fig. 17). Right panel: the distribution of p values for this random set, where the dashed and solid vertical lines indicate the observed p value (2.54×10^{-11}) at the IGC locus and the 0.05 cutoff, respectively.

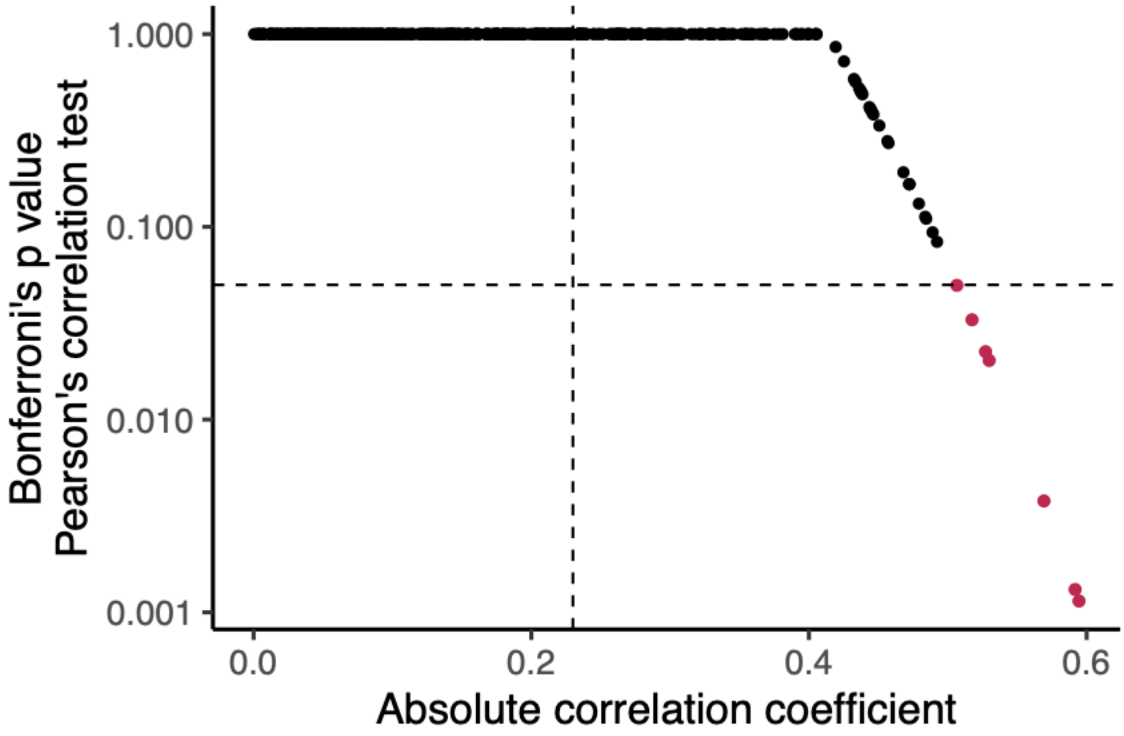

Supplementary Fig. 19. Correlation between paralog-specific *TCAF* CN at the putative IGC locus and latitudinal locations of human populations. Data were taken from Supplementary Fig. 18 and replotted with the absolute correlation coefficients and Bonferroni's p values (n=1,000 permutations of the dataset) for the randomly sampled loci. The seven loci that show absolute coefficients > 0.23 (the observed Pearson's correlation coefficient of 0.23 for the putative IGC acceptor locus in Supplementary Fig. 17) and with corrected p values < 0.05 are in red and are the most extreme. Note that none of the seven outliers have Pearson's correlation test p values (two-tailed) as small as the one observed in Supplementary Fig. 17 (Bonferroni's p value = 2.54e-8).

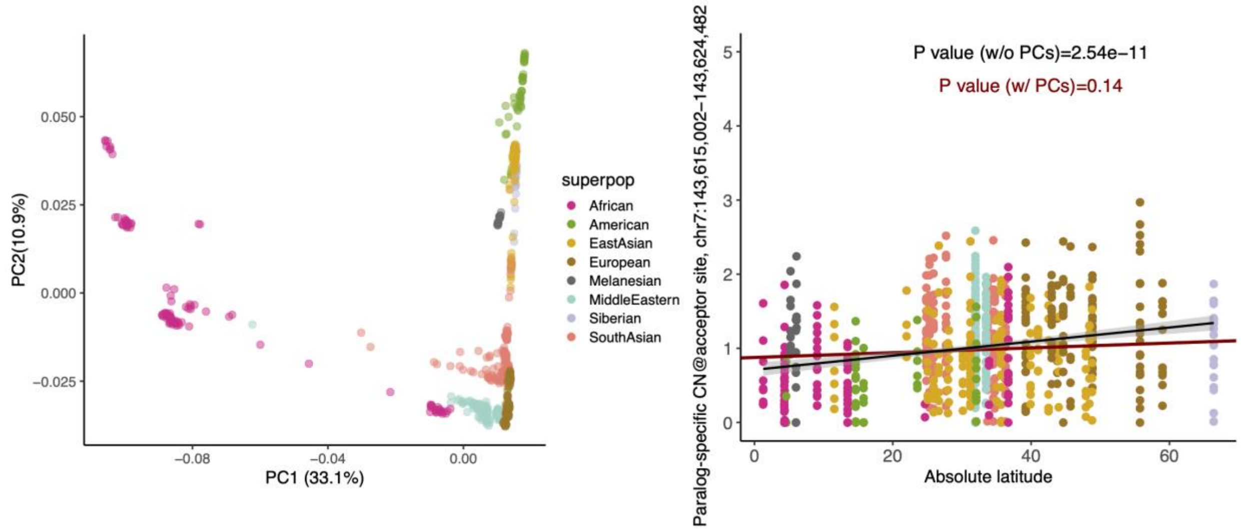

Supplementary Fig. 20. (Left) Principal component analysis (PCA) of the HGDP samples and (right) correlation between paralog-specific *TCAF* CN at the putative IGC locus and latitudinal locations of human populations with and without accounting for population structure using principal components (PCs). (Left) PCA was performed using 715,881 SNVs after linkage-disequilibrium pruning, and each circle is an individual colored by super-population groups. (Right) The black and red lines are the regression lines without and with PCs as covariates in the regression model. Pearson's correlation coefficient p values were computed across African (N=192 samples), Native American (N=75), East Asian (N=223), European (N=180), Melanesian (N=29), Middle Easterner (N=146), Siberian (N=45), and South Asian (N=205) individuals. Shaded area represents the 95% confidence interval of the fitted regression line.

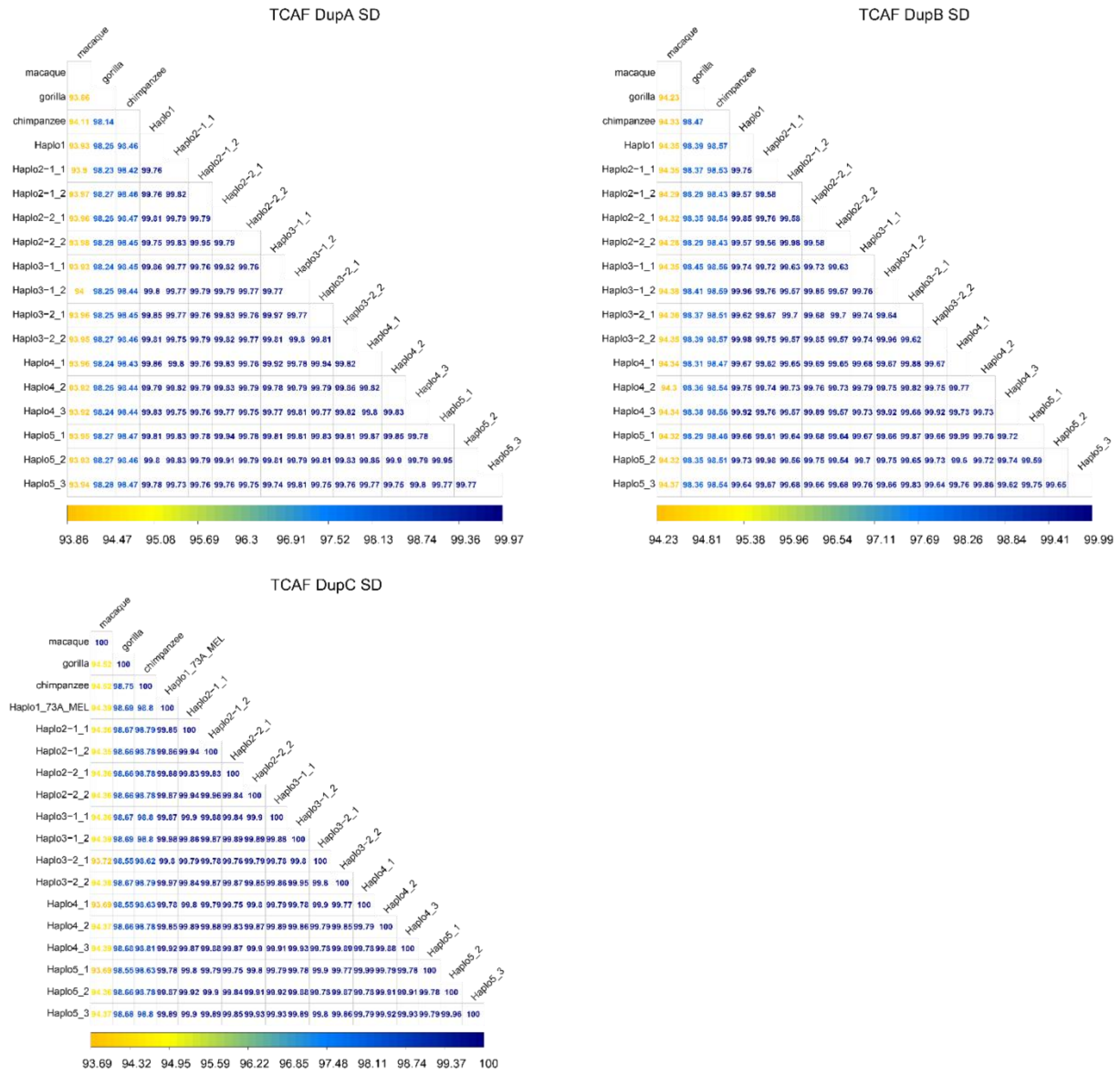

Supplementary Fig. 21. Pairwise sequence identity of *TCAF* SDs from the 15 BAC-assembled haplotypes reported in this study. Sequence identity was computed based on single base changes between two sequences and colored accordingly. For convenience, SDs on individual haplotypes were named from the centromeric to telomeric sides indicated by the last number after the underscore in the SD IDs. The coordinates of the SDs can be found in Supplementary Data 6.

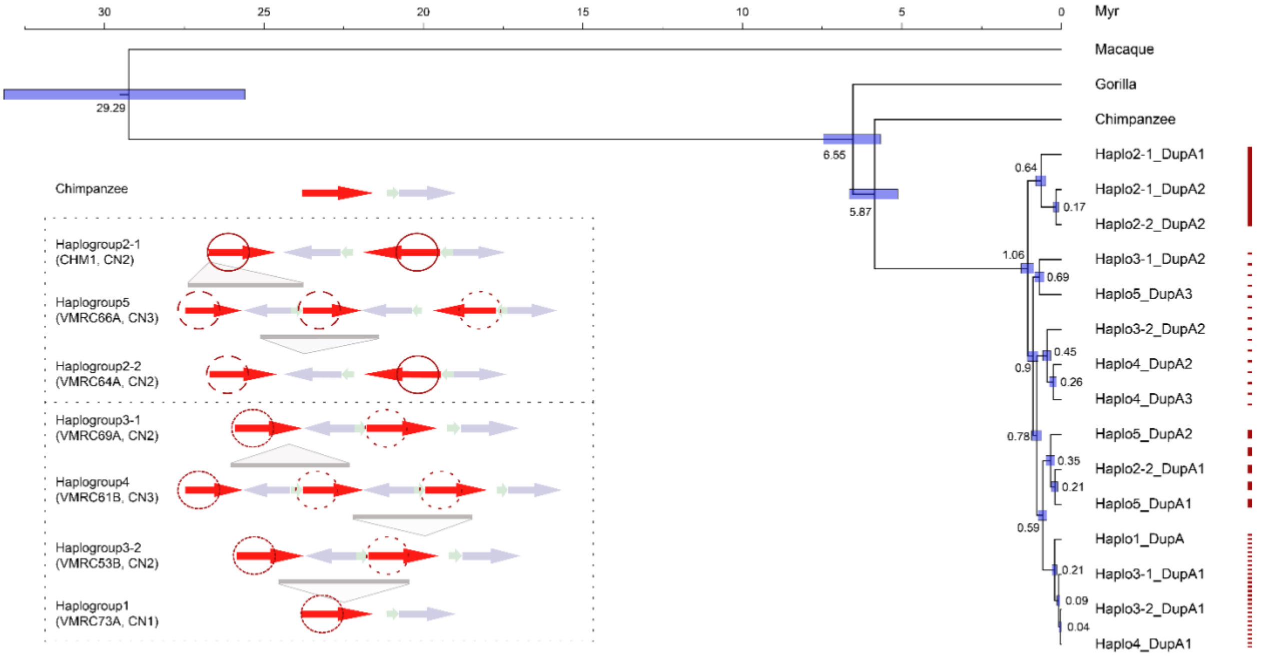

Supplementary Fig. 22. Phylogenetic reconstruction of the evolutionary history of *TCAF* SD DupA using BAC-assembled haplogroups. Phylogeny of the *TCAF* DupA sequences among haplogroups was inferred using BEAST (v.2.6.2) and five independent runs of 10 million iterations of Markov Chain Monte Carlo (Methods). Each number and horizontal bar at an internal node indicate the point estimate for the divergence (in million years, Myr) and its 95% highest posterior density interval, respectively. The inset shows the putative relationships among *TCAF* DupA sequences, where different types of circles correspond to groups annotated on the inferred phylogeny.

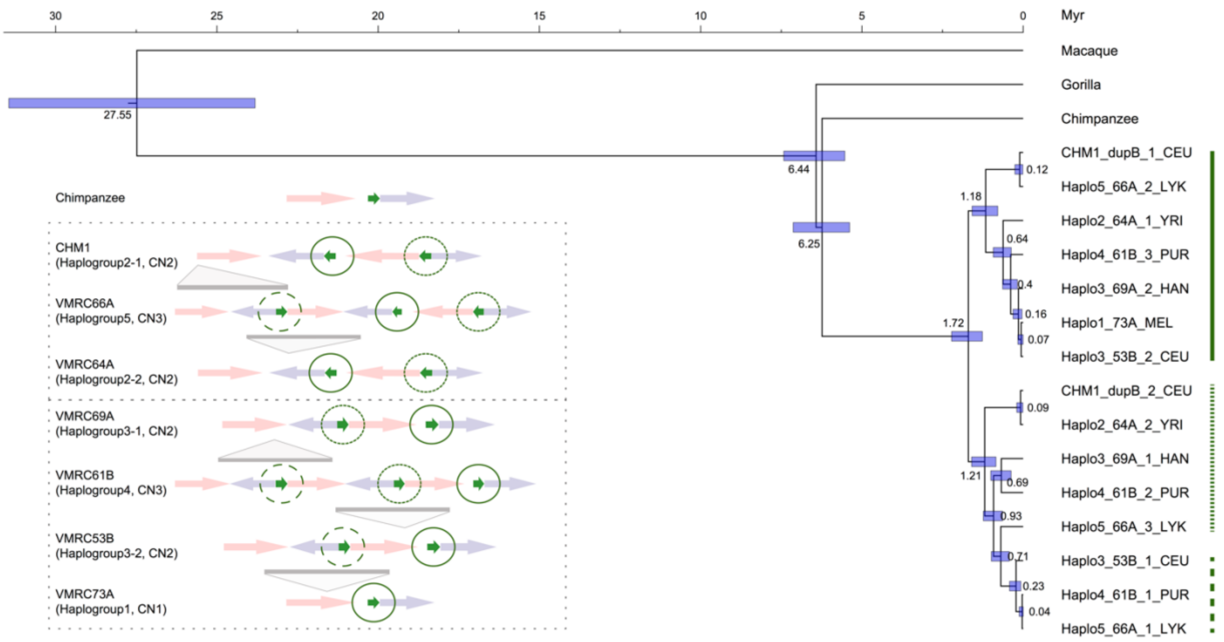

Supplementary Fig. 23. Phylogenetic reconstruction of the evolutionary history of *TCAF* SD DupB using BAC-assembled haplogroups. Phylogeny of the *TCAF* DupB sequences among haplogroups was inferred using BEAST (v.2.6.2) and five independent runs of 10 million iterations of Markov Chain Monte Carlo (Methods). Each number and horizontal bar at an internal node indicate the point estimate for the divergence (in million years) and its 95% highest posterior density interval, respectively. Branches with posterior probabilities <90% are colored in red. The inset shows the putative relationships among *TCAF* DupB sequences, where different types of circles correspond to groups annotated on the inferred phylogeny.

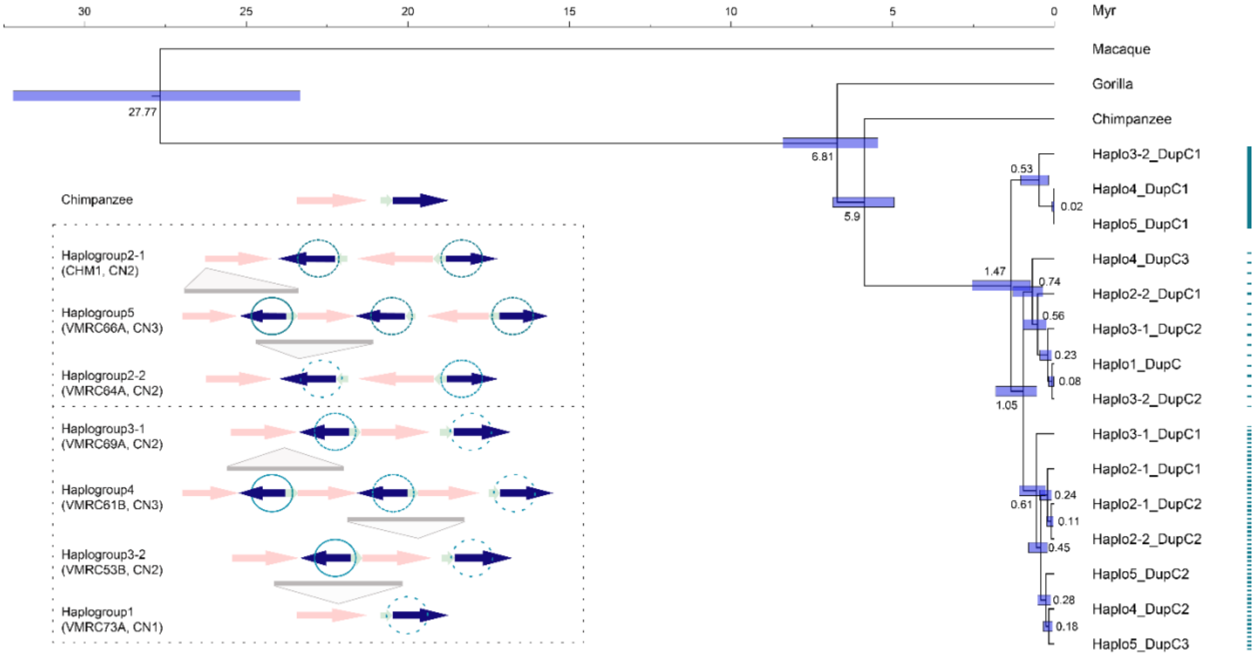

Supplementary Fig. 24. Phylogenetic reconstruction of the evolutionary history of *TCAF* SD DupC using BAC-assembled haplogroups. Phylogeny of the *TCAF* DupC sequences among haplogroups was inferred using BEAST (v.2.6.2) and five independent runs of 10 million iterations of Markov Chain Monte Carlo (Methods). Each number and horizontal bar at an internal node indicate the point estimate for the divergence (in million years) and its 95% highest posterior density interval, respectively. The inset shows the putative relationships among *TCAF* DupC sequences, where different types of circles correspond to groups annotated on the inferred phylogeny.

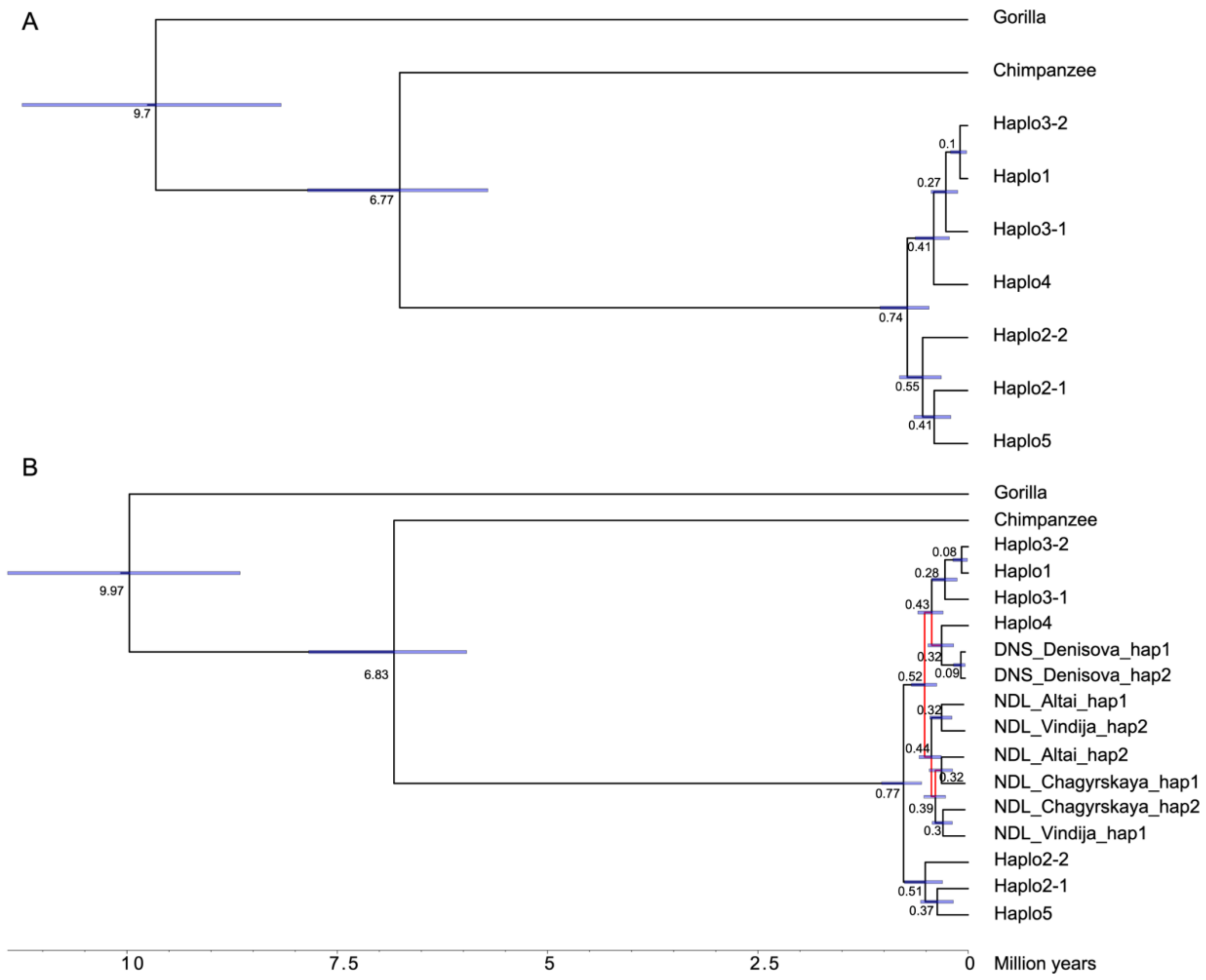

Supplementary Fig. 25. Reconstruction of phylogeny for the *TCAF* haplotypes using the 12.3 kbp single-copy unique sequences embedded within the *TCAF* SD region (Fig. 1). Phylogenetic inferences were performed using BEAST (v2.5; Methods). (A) Sequences from two nonhuman primate and seven human haplotypes. (B) The same sequences from the top panel and the archaic hominin haplotypes. The numbers are the point estimates for the branch times, while the purple bars show their 95% highest probability densities. Branches colored red have <90% posterior probability supports.

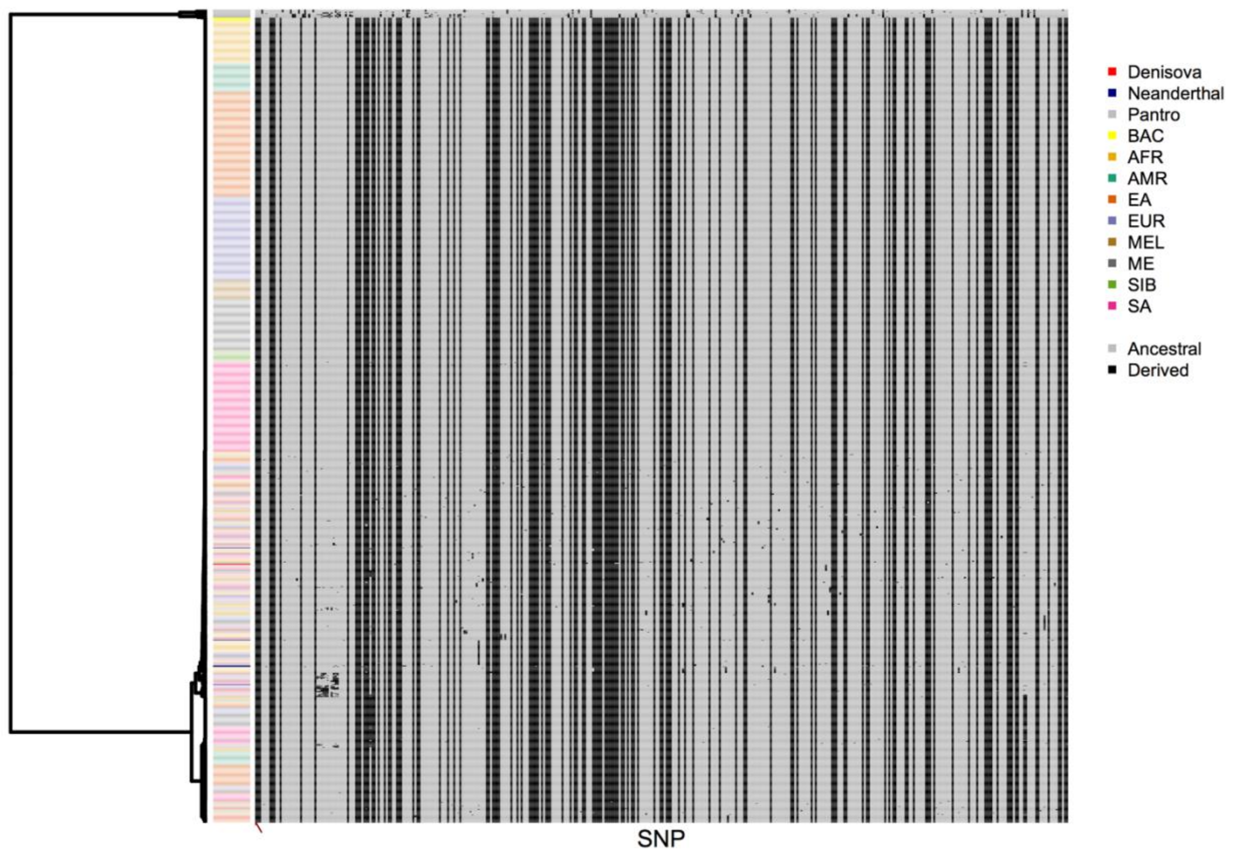

Supplementary Fig. 26. Haplotypes of the HGDP panel, four archaic hominins, and eight chimpanzee samples using the 428 SNVs from the single-copy unique region embedded with the *TCAF* SD region. The rows and columns are haplotypes and SNVs, respectively. A hierarchical clustering based on Ward's minimum variance method were performed. Note that the colors for all HGDP haplotypes were transparent, while those for the archaic and chimpanzee samples are solid.

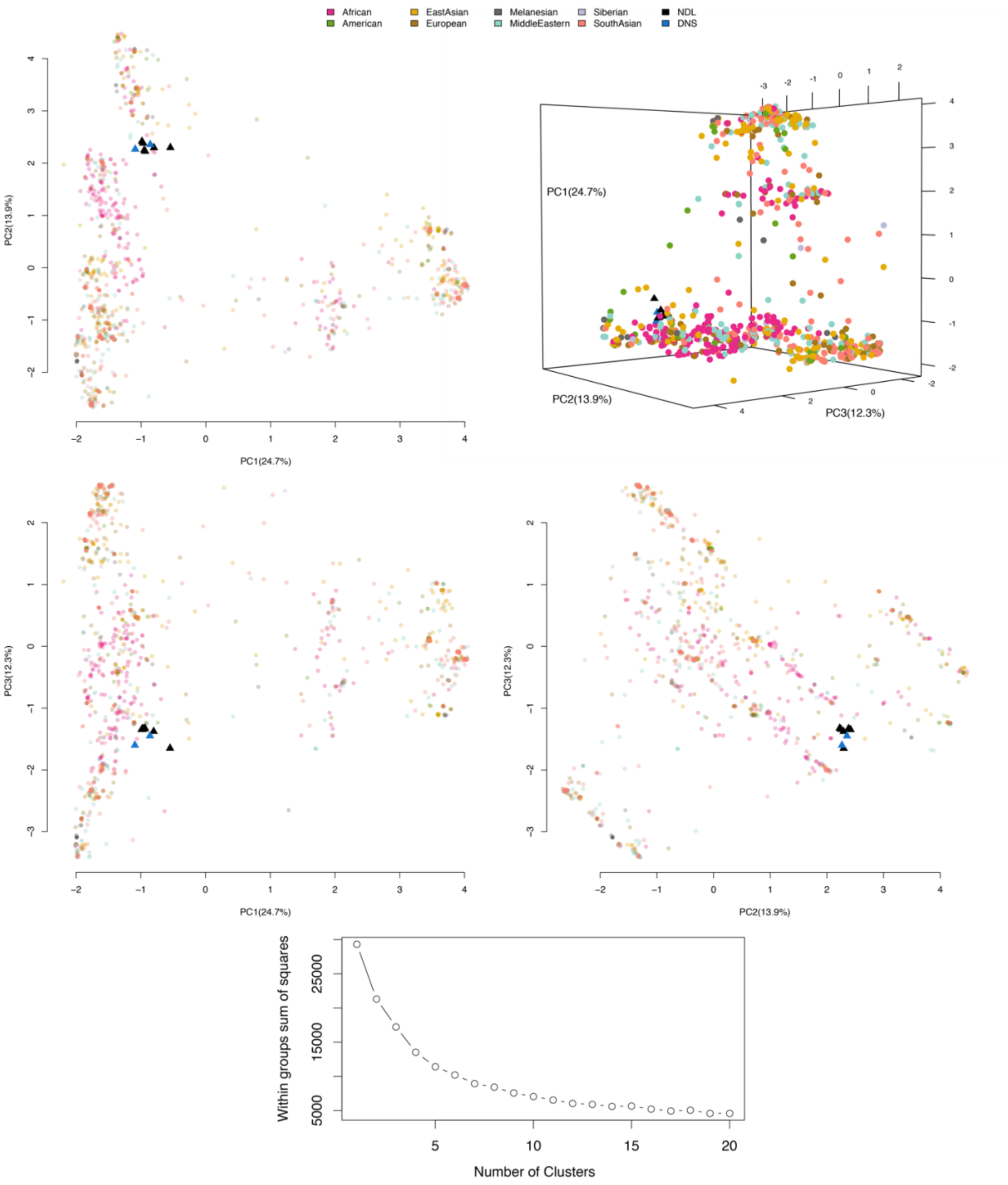

Supplementary Fig. 27. Haplotype-based PCA for 1,275 SNVs in the three single-copy unique loci at the *TCAF* SD region. Haplotypes were inferred computationally for the HGDP panel as well as the four archaic genomes (Methods). Each dot is a modern-day human haplotype, colored according to its population origin. Black and blue triangles are the Neanderthal and Denisovan haplotypes. Haplotype clusters were formed using an iterative approach based on the K-mean clustering technique. The best K (K=12) was determined by minimizing the sum of squares of within-groups distance (the bottom panel).

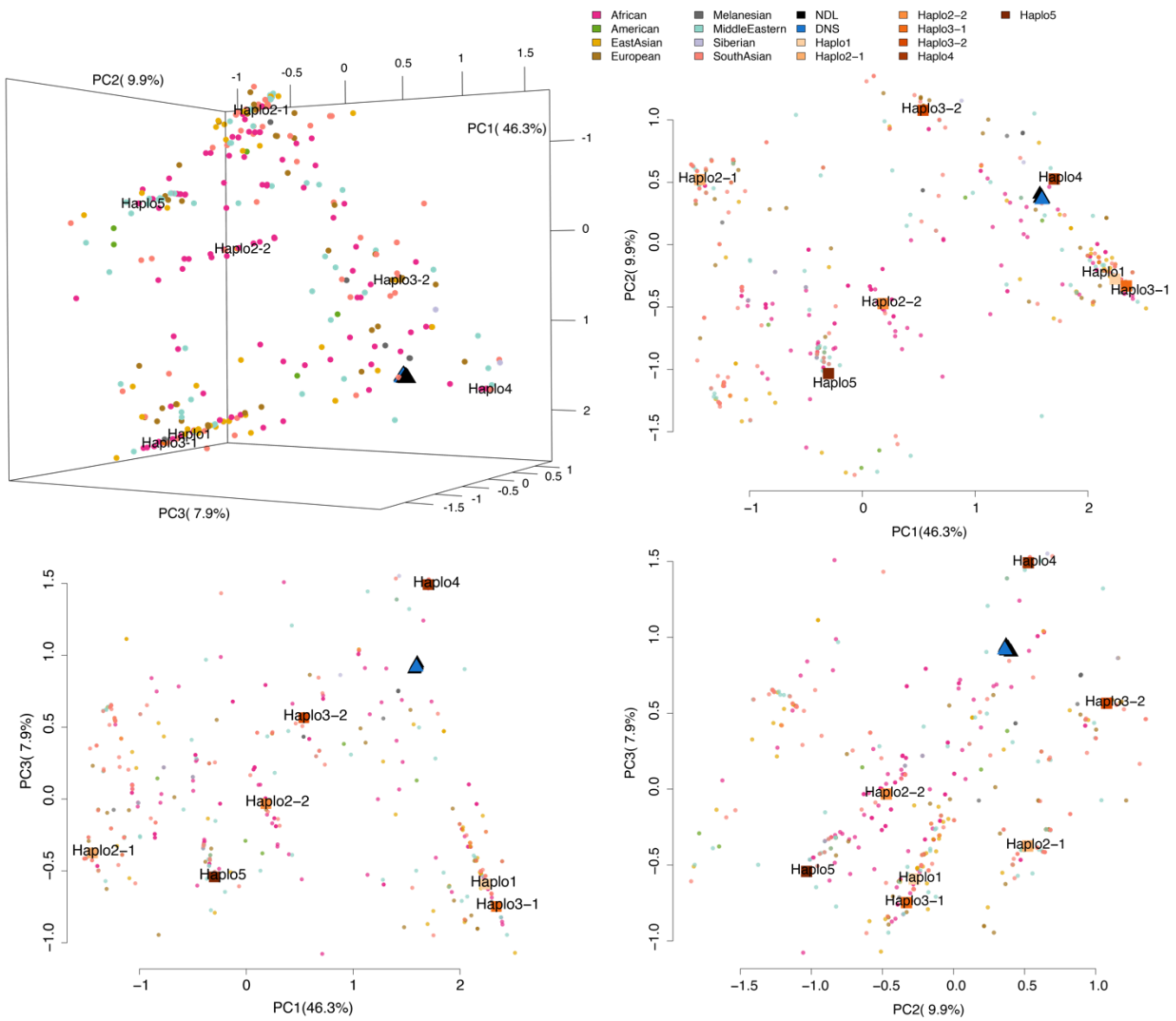

Supplementary Fig. 28. Haplotype-based PCA for 428 SNVs in the 12.3 kbp unique diploid sequence embedded in the *TCAF* SD region. Haplotypes were inferred computationally for the HGDP panel as well as the four archaic genomes (Methods). Each dot/triangle is a haplotype and colored according to its population origin.

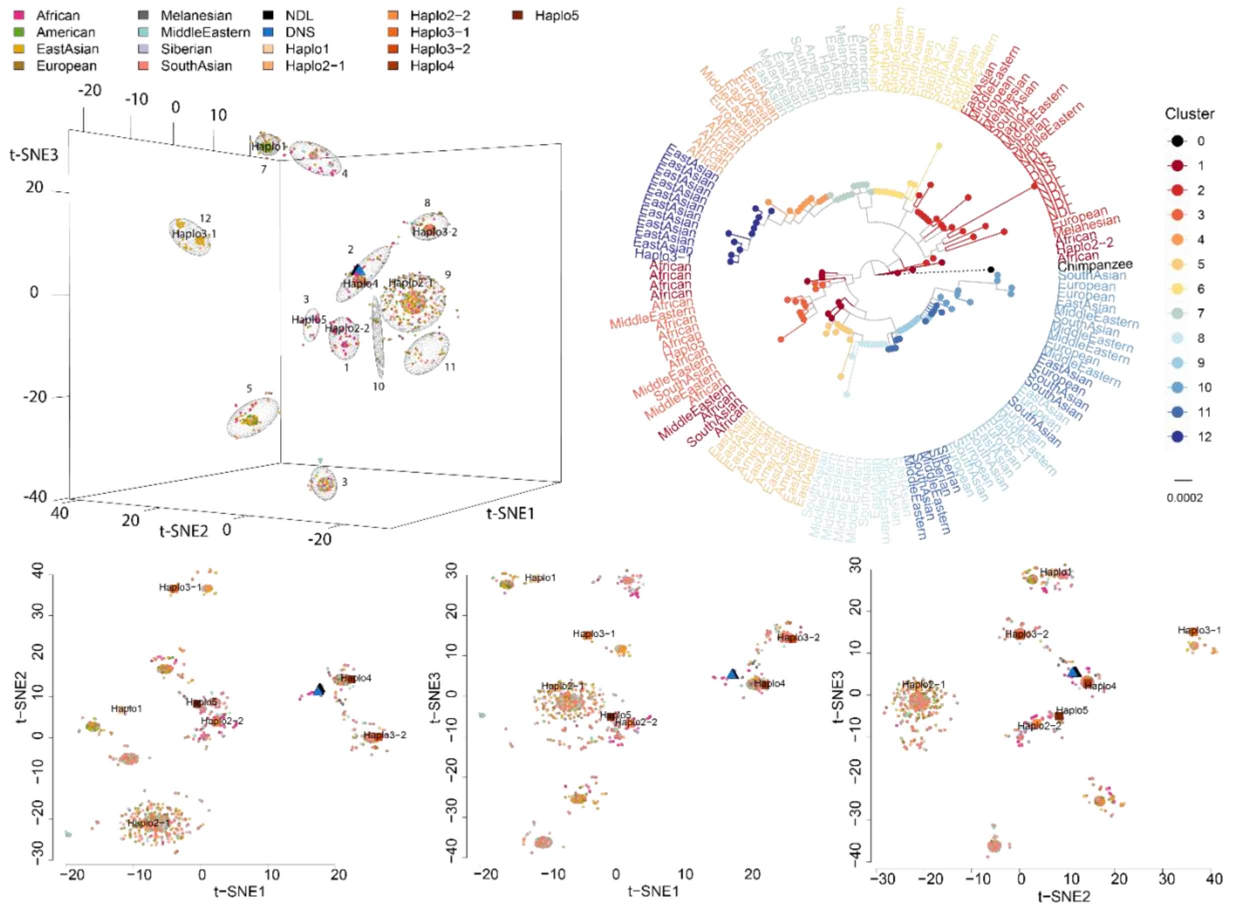

Supplementary Fig. 29. Rich haplotype diversity in humans at the 7q35 *TCAF* SD region using dimension-reduction and phylogenetic techniques. Haplotypes were inferred using 428 SNVs in the single-copy unique sequences embedded within the *TCAF* SD region (Fig. 1). Haplotype-based PCA was performed, followed by haplotype clustering and cluster visualization using the dimension-reduction technique, t-SNE (Methods). On the t-SNE plots, each dot/triangle is a haplotype and colored according to population origin. Numbers and ellipses in the 3D t-SNE plots indicate individual clusters**.** Sequences from the same region in the seven haplogroups are projected onto the plot and labelled. The maximum likelihood phylogeny was constructed using 10 randomly selected haplotypes from the 12 inferred clusters, in addition to eight archaic and one chimpanzee haplotypes. Note that the branch length of chimpanzee (dashed line) is truncated by 90% of its actual length for the purpose of illustration.

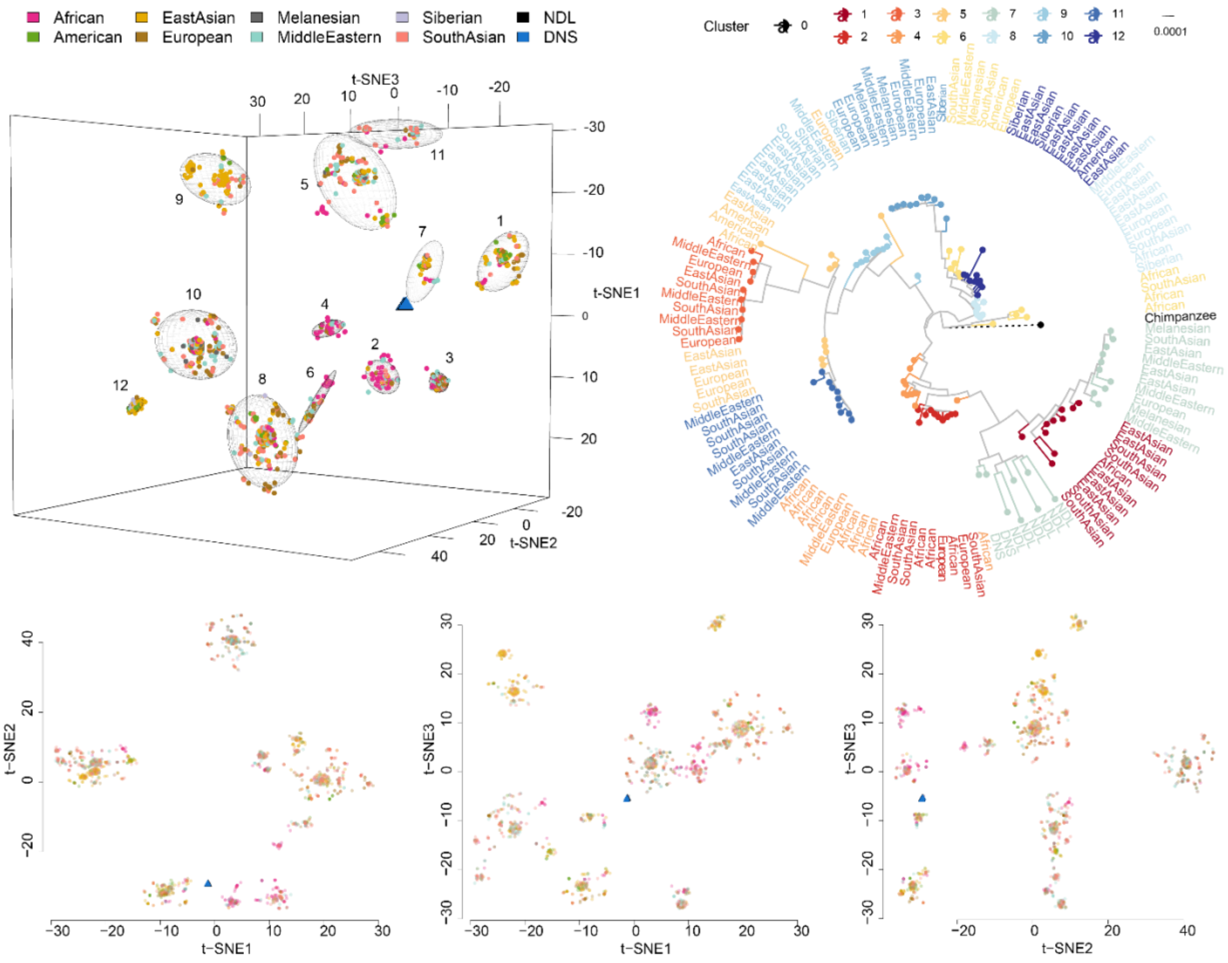

Supplementary Fig. 30. Rich haplotype diversity in humans at the 7q35 *TCAF* SD region using dimension-reduction and phylogenetic techniques. Haplotypes were inferred using 847 SNVs in the two single-copy unique sequences flanking the *TCAF* SD region (Fig. 1). Haplotype-based PCA was performed, followed by haplotype clustering and cluster visualization using the dimension-reduction technique, t-SNE (Methods). On the t-SNE plots, each dot/triangle is a haplotype and colored according to population origin. Numbers and ellipses in the 3D t-SNE plots indicate individual clusters. The maximum likelihood phylogeny was constructed using 10 randomly selected haplotypes from the 12 inferred clusters, in addition to eight archaic and one chimpanzee haplotypes. Note that the branch length of chimpanzee (dashed line) is truncated by 90% of its actual length for the purpose of illustration.

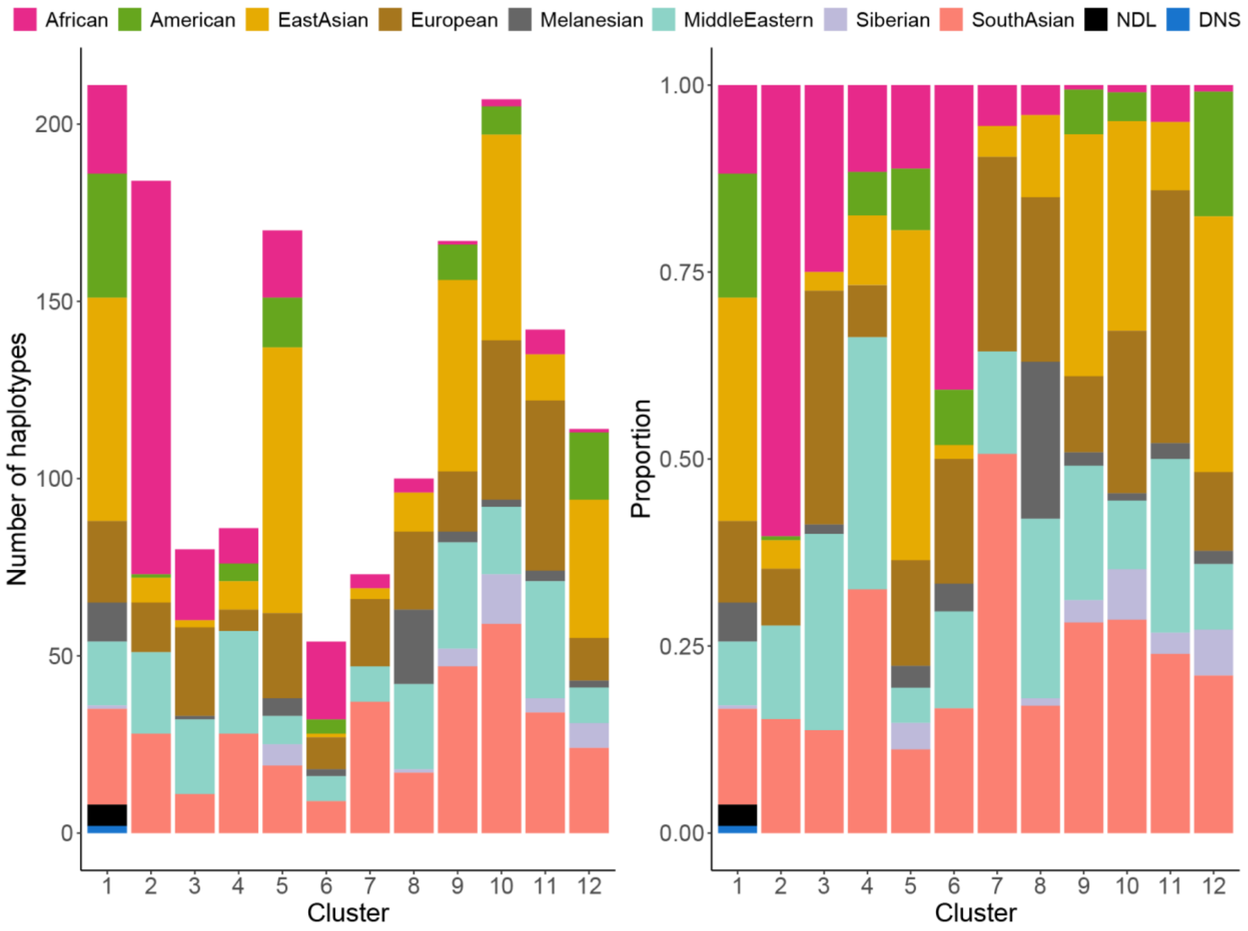

Supplementary Fig. 31. Population composition of *TCAF* haplotype clusters. Haplotypes of the modern (HGDP) and archaic (Neanderthal/NDL and Denisovan/DNS) samples were inferred using 1,275 SNVs from the 52.3 kbp sequences of the three unique diploid-copy regions (Fig. 1). Note that all archaic human haplotypes are assigned to cluster 1.

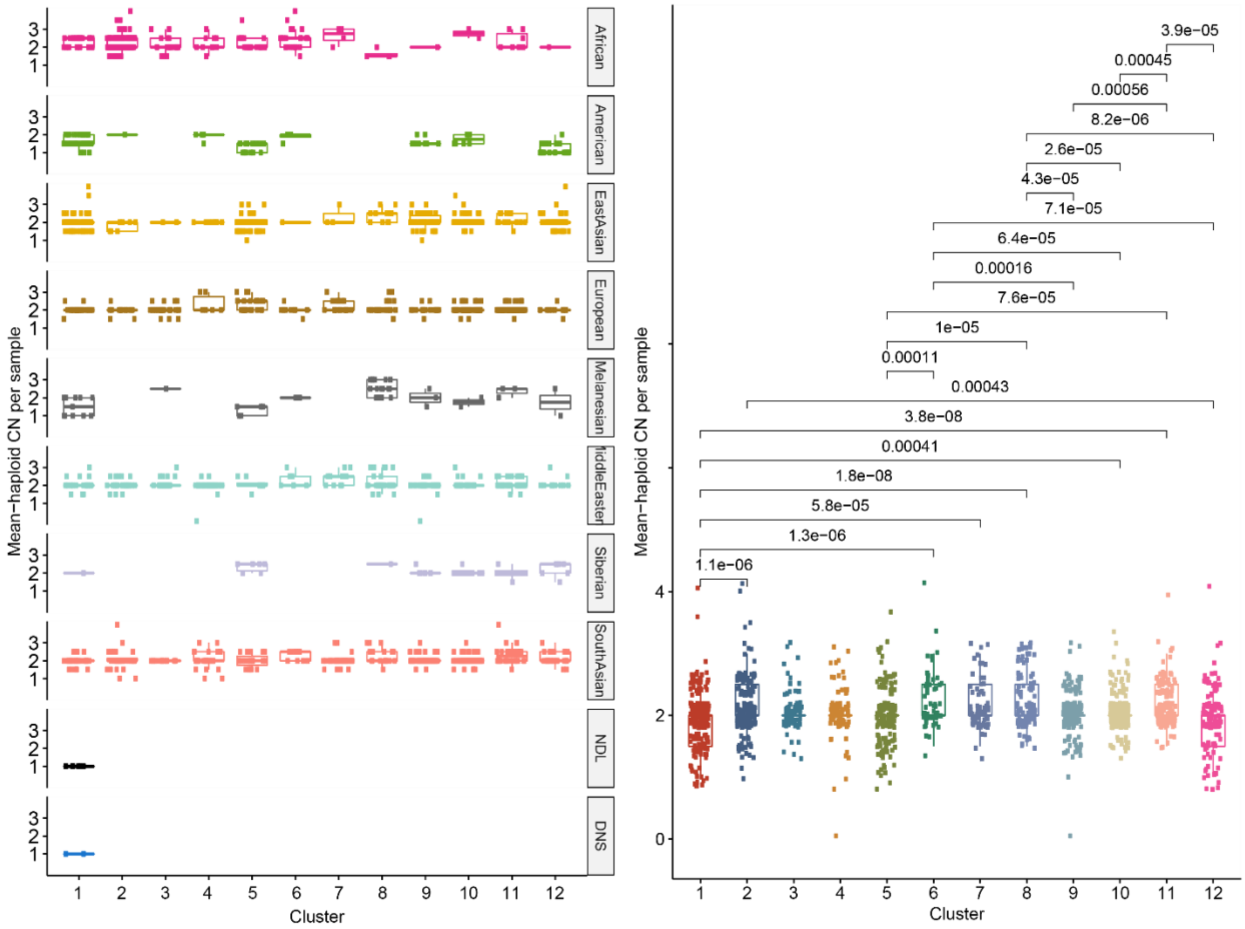

Supplementary Fig. 32. Significant difference in the per-sample mean-haploid *TCAF* CN among haplotype clusters. Haplotypes of the modern (HGDP) and archaic (Neanderthal/NDL and Denisovan/DNS) samples were inferred using 1,275 SNVs from the 52.3 kbp sequences of the three unique diploid-copy regions (Fig. 1). For each haplotype, the per-sample mean-haploid CN is the half of the overall diploid CN for a given sample. For each boxplot, the lower and upper hinges correspond to the first and third quartiles, respectively, in addition to the median value. The upper and lower whiskers show 1.5 * IQR from the upper and lower hinges, respectively, where IQR is the inter-quartile range defined as the distance between the first and third quartiles. Significance levels were computed using the Mann-Whitney *U* test (two-tailed) among clusters (Cluster 1: 211 haplotypes, Cluster 2: 184 haplotypes, Cluster 3: 80 haplotypes, Cluster 4: 86 haplotypes, Cluster 5: 170 haplotypes, Cluster 6: 54 haplotypes, Cluster 7: 73 haplotypes, Cluster 8: 100 haplotypes, Cluster 9: 167 haplotypes, Cluster 10: 207 haplotypes, Cluster 11: 142 haplotypes, and Cluster 12: 114 haplotypes). Only p values that survive Bonferroni correction are listed on the plot.

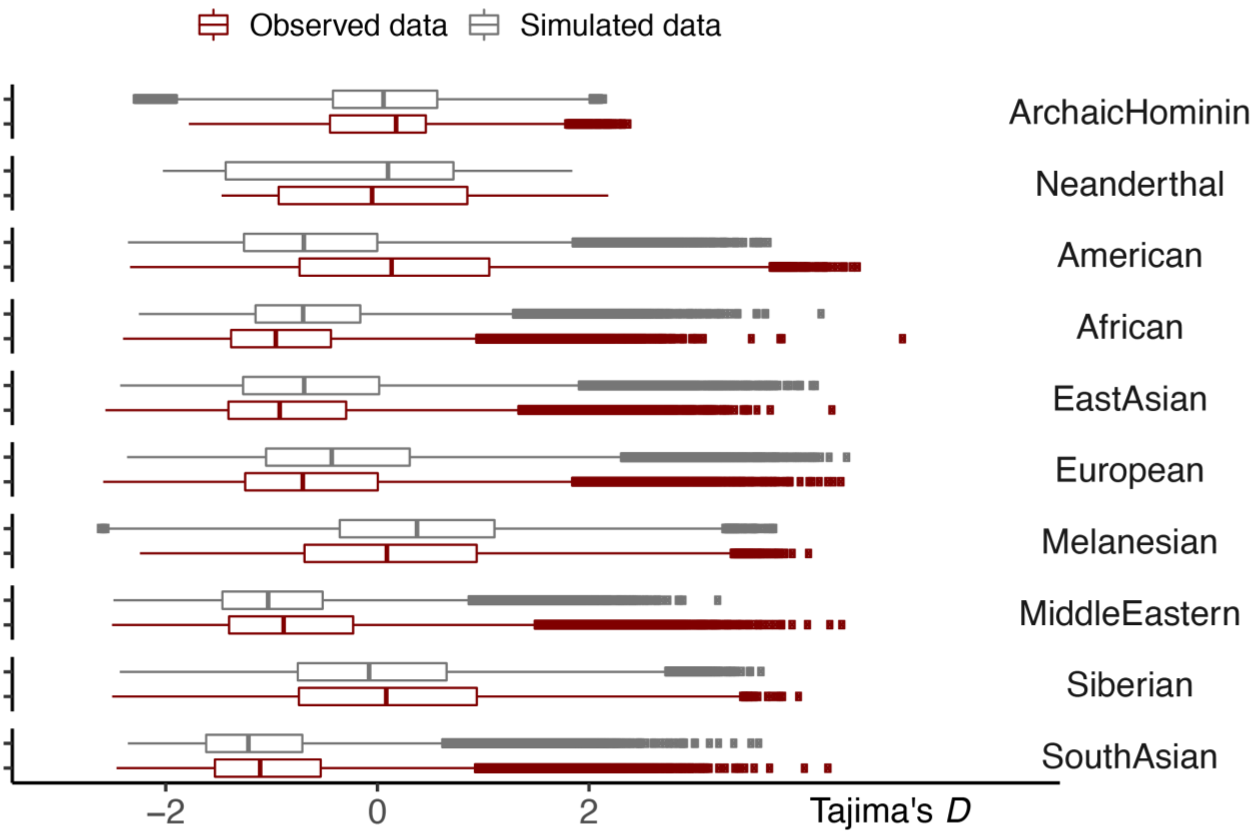

Supplementary Fig. 33. Comparisons of genome-wide Tajima's *D* statistic values between observed and simulated data using models listed in Supplementary Data 9. Tajima's *D* values are computed using the same 2000 bp windows across the genome (n=1,224,349 windows) as described in the main text. For each boxplot, the lower and upper hinges correspond to the first and third quartiles, respectively, in addition to the median value. The upper and lower whiskers show 1.5 * IQR from the upper and lower hinges, respectively, where IQR is the inter-quartile range defined as the distance between the first and third quartiles.

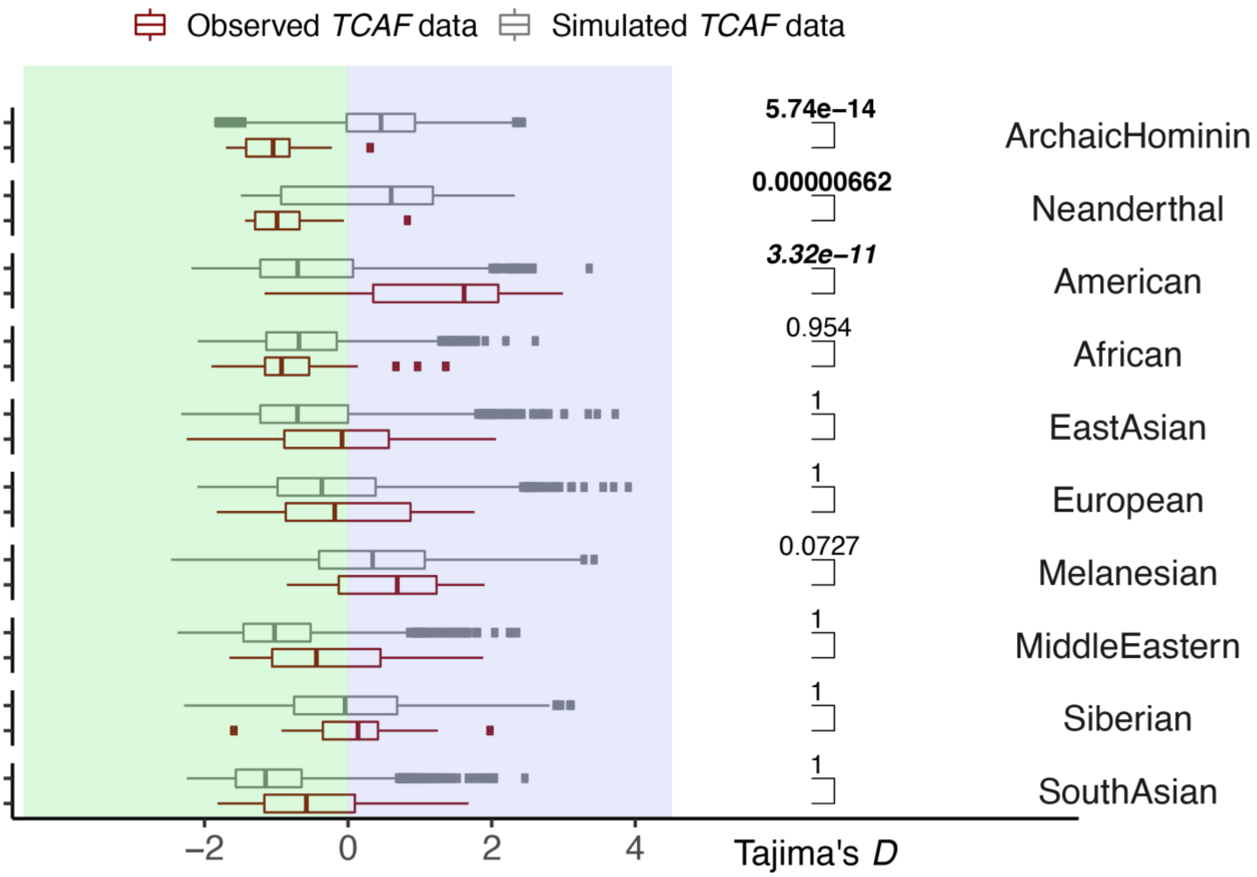

Supplementary Fig. 34. Comparisons of Tajima's *D* statistic values between observed and simulated *TCAF* data using models listed in Supplementary Data 9. Tajima's *D* values were computed using 2000 bp windows (n=29 windows) as described in the main text. For each boxplot, the lower and upper hinges correspond to the first and third quartiles, respectively, in addition to the median value. The upper and lower whiskers show 1.5 * IQR from the upper and lower hinges, respectively, where IQR is the inter-quartile range defined as the distance between the first and third quartiles. P values (one-tailed) were computed by testing if the observed values are significantly more negative than those from simulated data, except for the American and Melanesian groups, where their p values represent if the observed Tajima's *D* values are significantly more positive (*italic*) than those from the simulated data. Asterisks indicate Bonferroni's p values < 0.05.

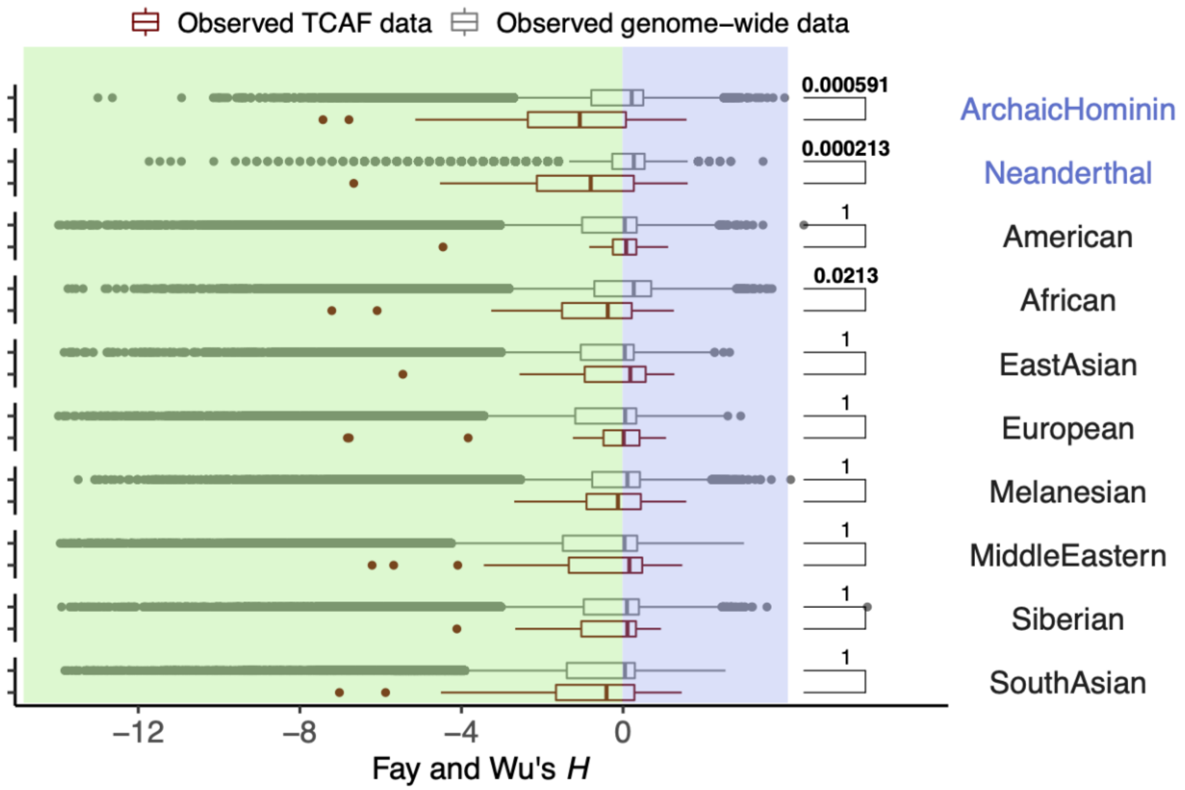

Supplementary Fig. 35. Comparisons of Fay and Wu's *H* values between observed *TCAF* and genome-wide data. Fay and Wu's *H* values were computed using 2000 bp windows as described in the main text. Bonferroni's p values (one-tailed) were computed by testing if the observed values at *TCAF* (n=29 windows) are significantly more negative (green) than those from the genome-wide data (n=1,224,349 windows). For each boxplot, the lower and upper hinges correspond to the first and third quartiles, respectively, in addition to the median value. The upper and lower whiskers show 1.5 * IQR from the upper and lower hinges, respectively, where IQR is the inter-quartile range defined as the distance between the first and third quartiles. Bold fonts show Bonferroni's p values (one-tailed) < 0.05. Note that only the Neanderthal and archaic hominin groups (blue IDs) have significantly negative Tajima's *D* values (Supplementary Fig. 34).

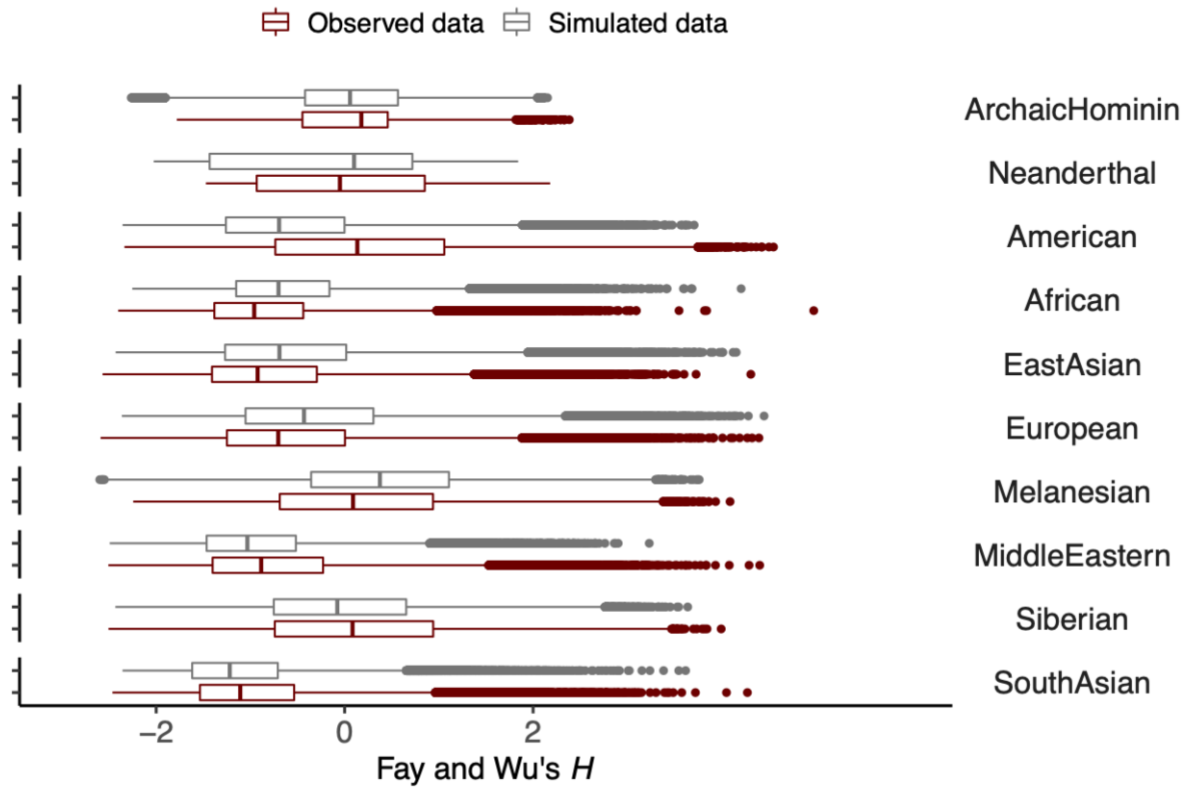

Supplementary Fig. 36. Comparisons of genome-wide Fay and Wu's *H* statistic values between observed and simulated data using models listed in Supplementary Data 9. Fay and Wu's *H* values are computed using the same 2000 bp windows (n=1,224,349 windows across the genome) across the genome as described in the main text. For each boxplot, the lower and upper hinges correspond to the first and third quartiles, respectively, in addition to the median value. The upper and lower whiskers show 1.5 * IQR from the upper and lower hinges, respectively, where IQR is the inter-quartile range defined as the distance between the first and third quartiles.

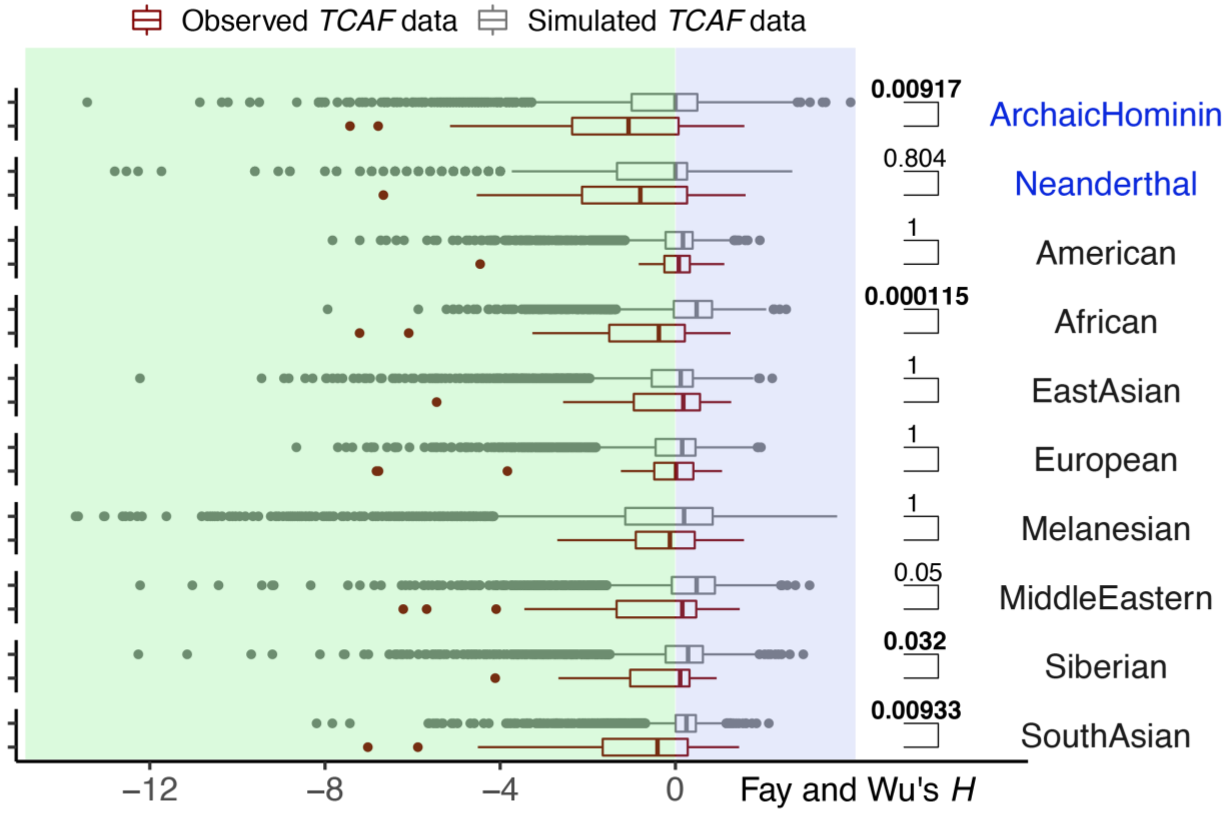

Supplementary Fig. 37. Comparisons of Fay and Wu's *H* values between observed and simulated *TCAF* data using models listed in Supplementary Data 9. Fay and Wu's *H* values were computed using 2000 bp windows (n=29 windows) as described in the main text. Bonferroni's p values (one-tailed) were computed by testing if the observed values are significantly more negative (green) than those from simulated data. For each boxplot, the lower and upper hinges correspond to the first and third quartiles, respectively, in addition to the median value. The upper and lower whiskers show 1.5 * IQR from the upper and lower hinges, respectively, where IQR is the inter-quartile range defined as the distance between the first and third quartiles. Bold fonts show Bonferroni's p values (one-tailed) < 0.05. Note that only the Neanderthal and archaic hominin groups (blue IDs) have significantly negative Tajima's *D* values (Supplementary Fig. 34).

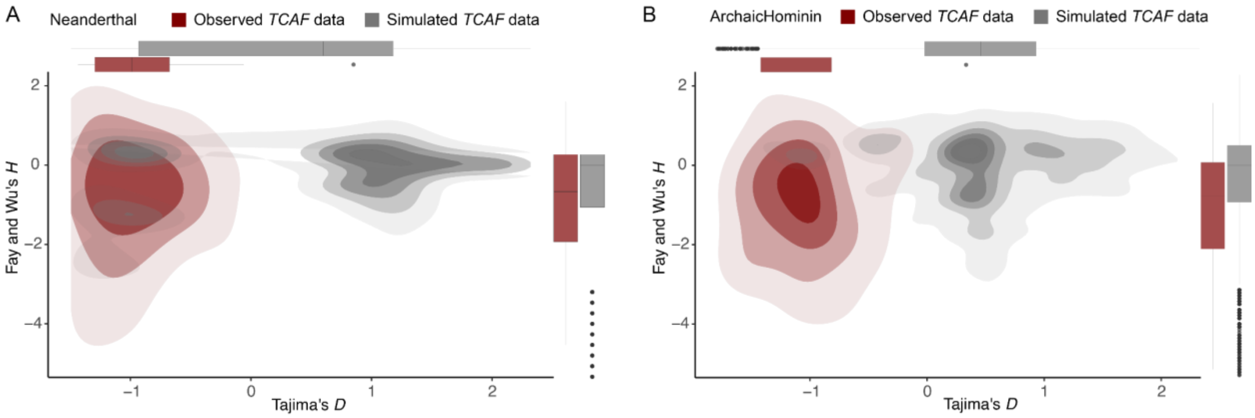

Supplementary Fig. 38. **Two-dimensional joint density plot showing distinct patterns of Tajima's *D* and Fay and Wu's *H* values between observed and simulated *TCAF* data for (A) Neanderthal group (n=3) and (B) archaic samples (n=4)**. Simulations were generated using the model from Jacob et al. (2019) listed in Supplementary Data 9.

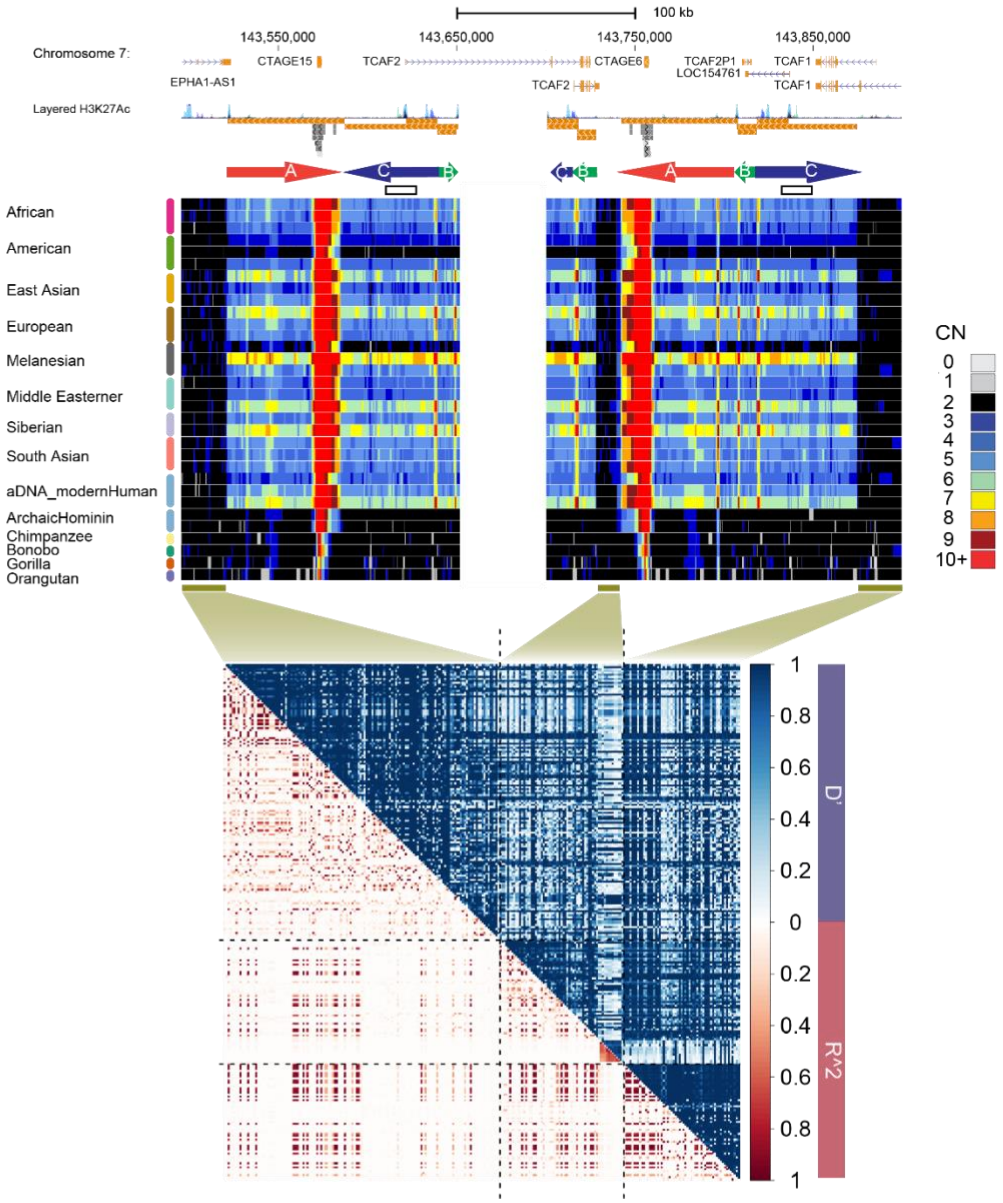

Supplementary Fig. 39. Marked effect of the inversion polymorphism between super haplogroups H4 and H5 on linkage disequilibrium patterns in hominins. Top panel: the browser shot at *TCAF* locus with SD, gene, and CN annotations (see Fig. 1 for more details). Bottom panel: patterns of linkage disequilibrium among the three unique diploid regions (dark green boxes) around the *TCAF* locus were inferred using D' (blue) and R^2 (red) and 263 SNVs that have minor allele frequency > 1%.

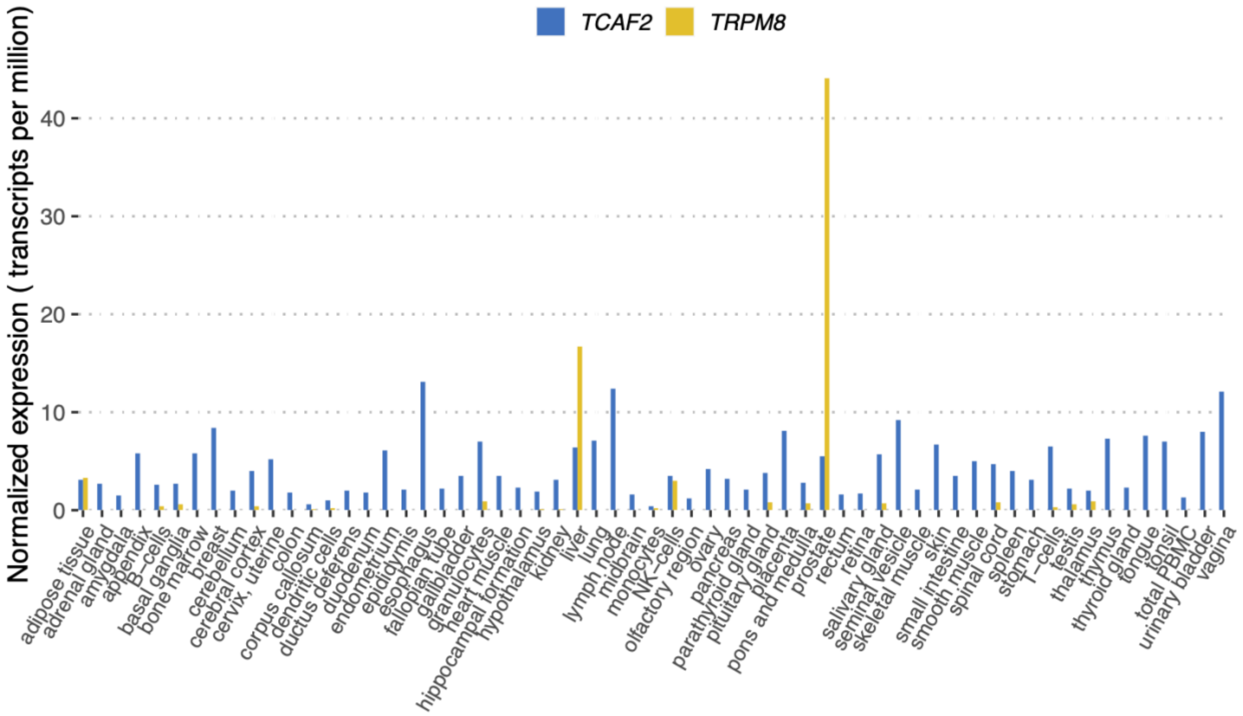

Supplementary Fig. 40. Normalized gene expression for *TCAF2* and *TRPM8* across 55 tissues and 6 blood types using the consensus dataset in the Human Protein Atlas database (https://www.proteinatlas.org).

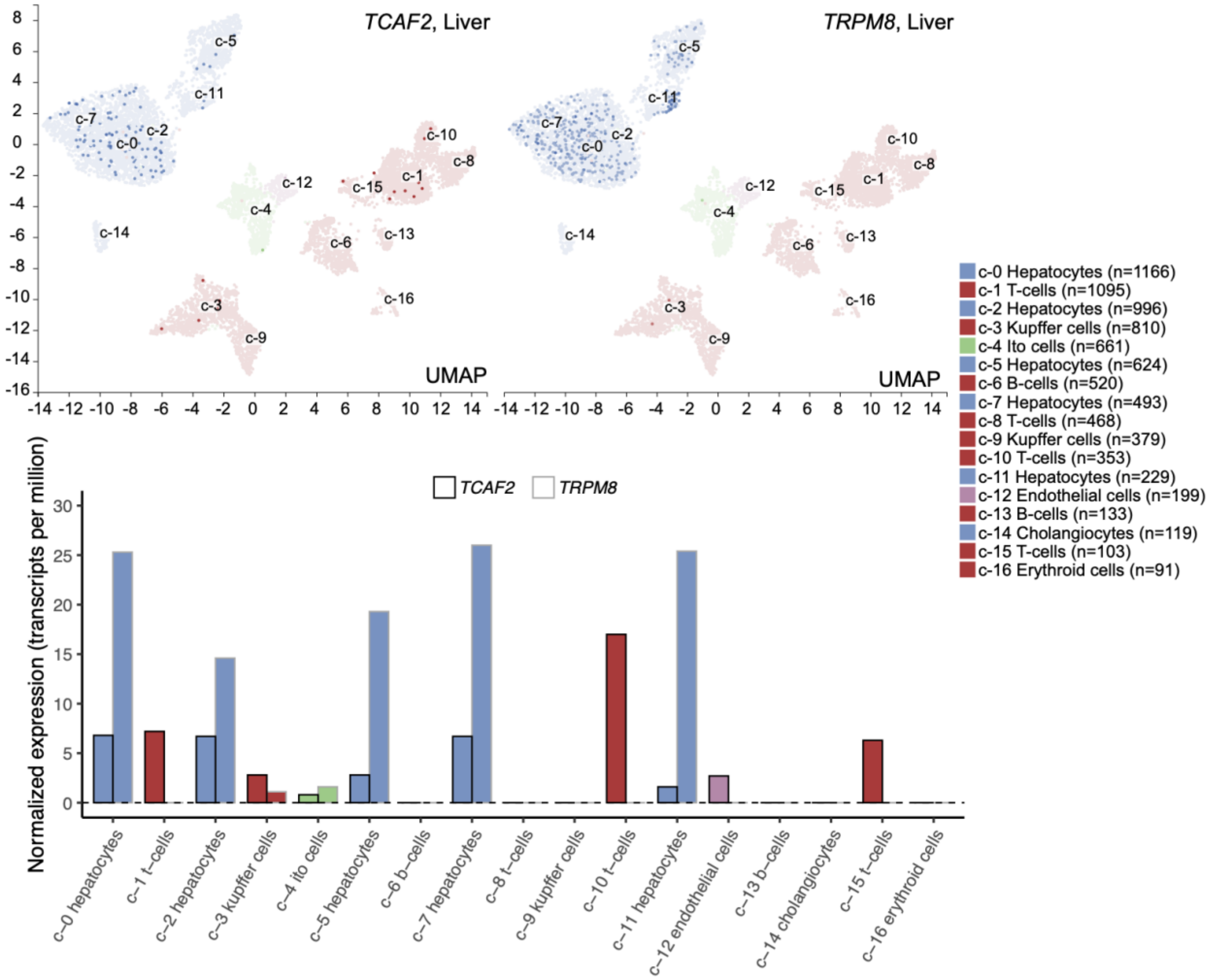

Supplementary Fig. 41. RNA expression of *TCAF2* (left) and *TRPM8* (right) in the single-cell-type clusters identified in the liver tissue visualized by a UMAP plot (top) and a bar chart (bottom) using the Human Protein Atlas database (HPA; https://www.proteinatlas.org/). Top: UMAP plot visualizes the cells in each cluster (c-0 to c-16); where each dot corresponds to a cell. Color is based on cell-type groups used in the Cell Type Atlas, and color intensity represents the individual cells according to % of max (log2(read_count+1)/log2(max(read_count)+1)*100) in five different bins (<1%, <25%, <50%, <75%, and ≥75%). Bottom: The bar chart shows normalized RNA expression in each cell-type cluster. Color-coding follows those on the top of the plot (i.e., cell type). Black and gray outlines of the bars indicate the *TCAF2* and *TRPM8* expression, respectively.

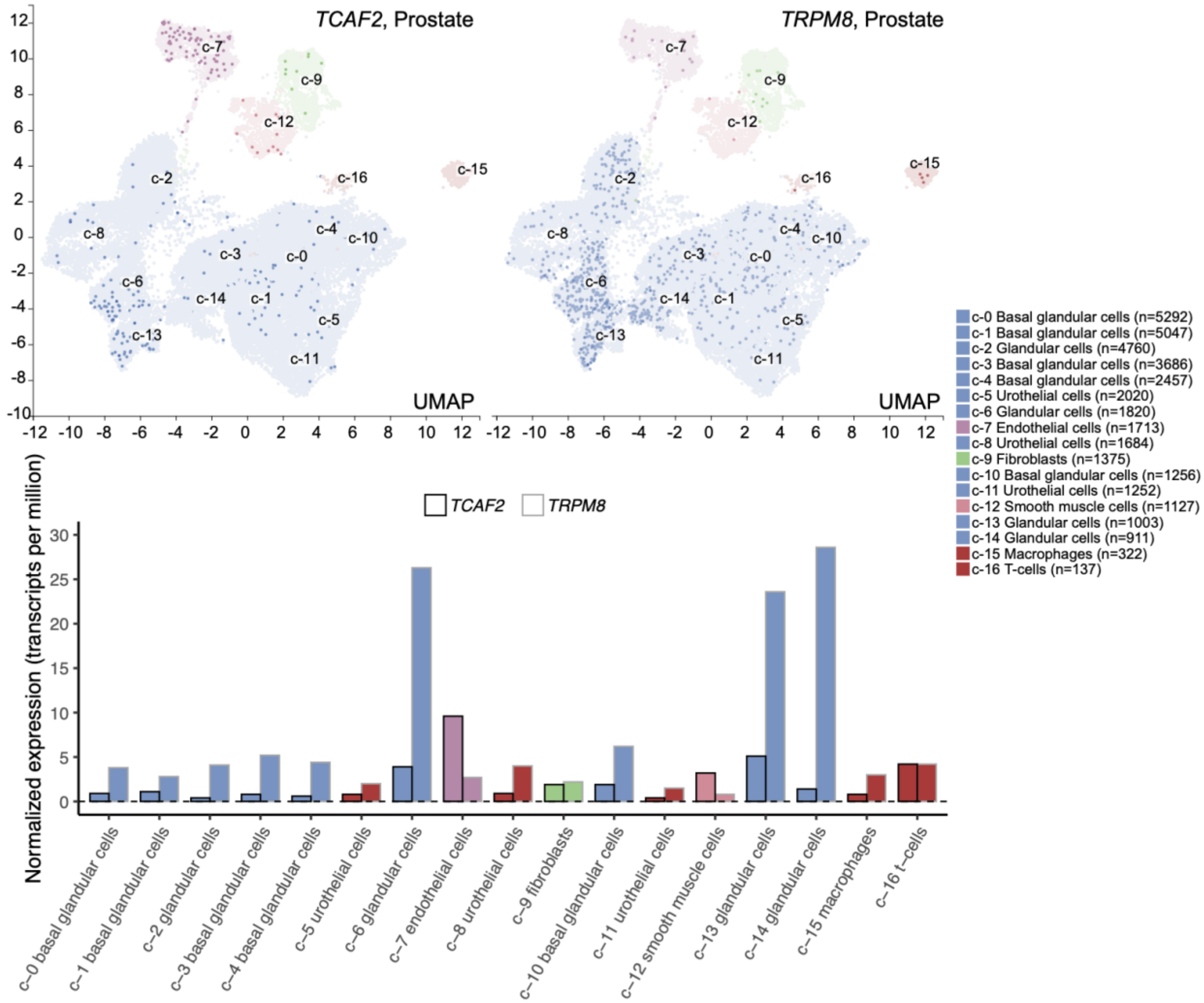

Supplementary Fig. 42. RNA expression of *TCAF2* (left) and *TRPM8* (right) in the single-cell-type clusters identified in the prostate tissue visualized by a UMAP plot (top) and a bar chart (bottom) using the Human Protein Atlas database (https://www.proteinatlas.org/). Top: UMAP plot visualizes the cells in each cluster (c-0 to c-16); where each dot corresponds to a cell. Color is based on cell-type groups used in the Cell Type Atlas, and color intensity represents the individual cells according to % of max (log2(read_count+1)/log2(max(read_count)+1)*100) in five different bins (<1%, <25%, <50%, <75%, and ≥75%). Bottom: The bar chart shows normalized RNA expression in each cell-type cluster. Color-coding follows those on the top of the plot (i.e., cell type). Black and gray outlines of the bars indicate the *TCAF2* and *TRPM8* expression, respectively.

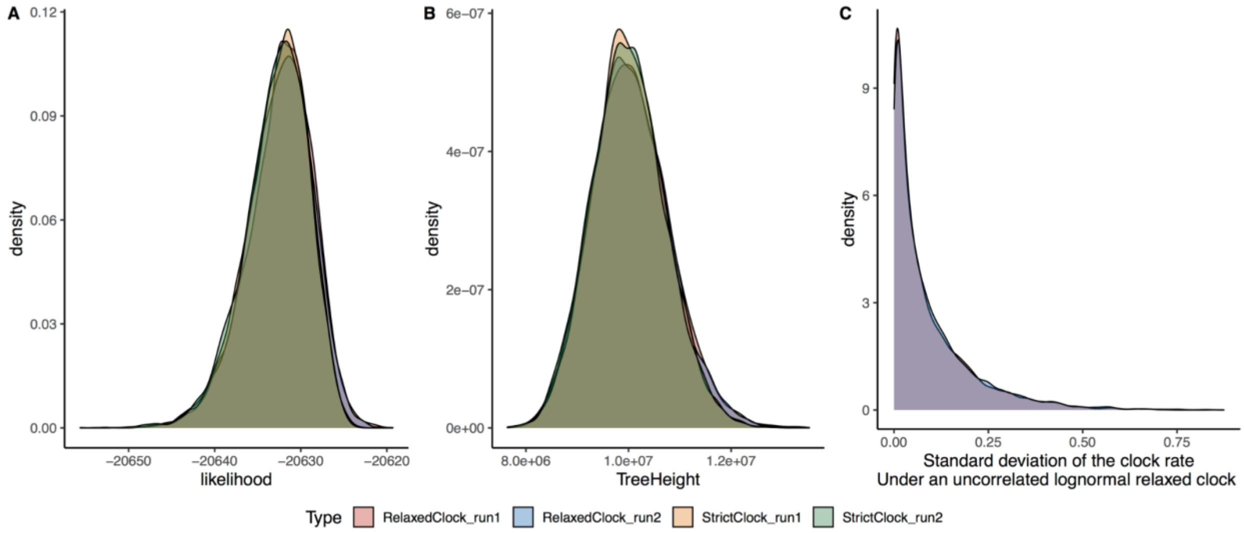

Supplementary Fig. 43. Comparisons of key statistics between models of an uncorrelated log-normal relaxed clock and a strict clock. For each model, two replicates were performed (run 1 and run 2). (A) Density of likelihood, (B) density of tree height, and (C) density of standard deviation for clock rate under an uncorrelated log-normal relaxed clock model. Note that the distributions of the standard deviation of clock rates under the relaxed clock model overlap with zero (i.e., no rate variation).

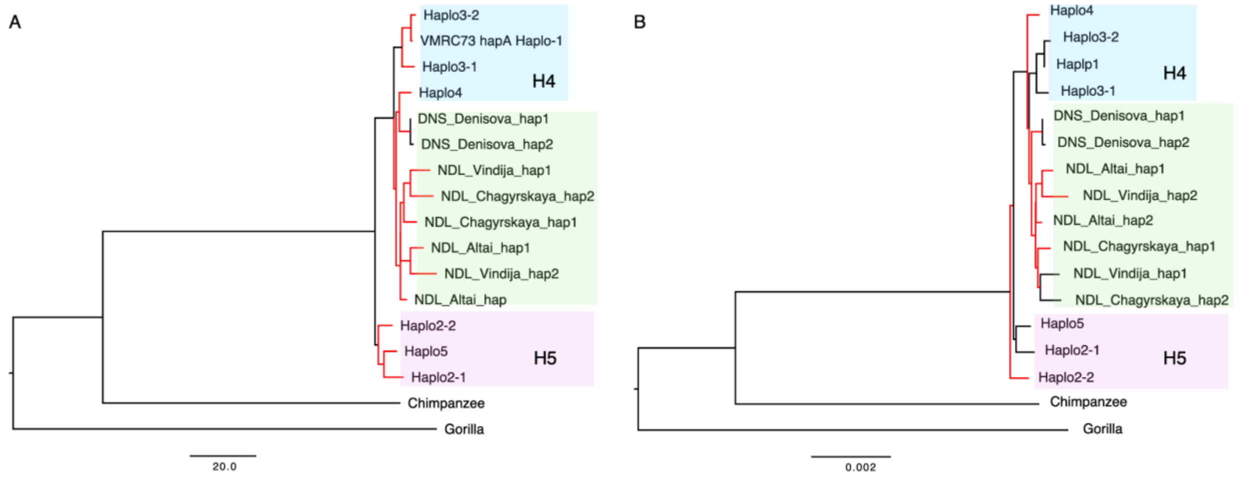

Supplementary Fig. 44. Inferred phylogenies of the modern human and archaic hominin haplotypes using the 12 kbp unique sequences embedded within *TCAF* SDs. Phylogenies were inferred using (A) maximum parsimony and (B) neighbor-joining methods, implemented in MEGA X (v11.0). Haplotypes are the same as in Fig. 4b from the main text. Branches with posterior probabilities <90% are colored in red. The branch length of the maximum parsimony tree refers to the number of substitutions per unit length, while that of the neighbor-joining tree is the number of substitutions per site for a given length unit.
