# Supplementary material for: Evidence for opposing selective forces operating on human-specific duplicated TCAF genes in Neanderthals and humans
Source: Nat Commun. 2021 Aug 25;12:5118. doi: 10.1038/s41467-021-25435-4 (PMC8387397; doi:10.1038/s41467-021-25435-4)
Supplement: Supplementary file 3 — Description of Additional Supplementary Files [file 41467_2021_25435_MOESM3_ESM.pdf]

Description of Additional Supplementary Files

Description: The supplementary data files contain all following Supplementary Data.

Supplementary Data 1. Pairwise comparisons in overall *TCAF* copy number between continental population groups.

Supplementary Data 2. Summary of *TCAF* contigs from four recently published long-read assembly studies. *TCAF* contigs were identified for each individual assembly by mapping contigs to the human reference genome GRCh38 using minimap2 (v2.17-r941). Despite some contigs spanning over the *TCAF* segmental duplications (SDs), in all cases the diploid assemblies fail to create contiguous sequences for both haplotypes.

Supplementary Data 3. Recurrent structural mutation events facilitating haplotype diversity in modern humans at the *TCAF* locus on Chromosome 7. Six different mutation events contribute to the diversity of the sequence structure at the *TCAF* locus illustrated in Fig. 2 (see also Supplementary Fig. 5). Breakpoints were inferred using pairwise sequence alignment-based smoothing to identify sudden disruptions in sequence identity (Fig. 2). Also reported are the length and sequence identity for the longest, high-identity sequences around the inferred breakpoints, which likely gave rise to non-allelic homologous recombination-mediated structural rearrangement events. RepeatMasker (v4.0.8) identifies repeat and/or transposable elements within the putative breakpoint locations.

Supplementary Data 4. Summary of unique full-length non-chimeric (FLNC) reads from seven tissue types using the PacBio Iso-Seq technology. For any given tissue, the number of FLNC reads per haplogroup is the count of transcripts that are the best and also uniquely aligned. Note that this is different from the later analysis of gene model discovery, where we allowed FLNC reads mapped to multiple haplotypes as long as the sequence identity differences for those secondary alignments is within 0.1% compared to the primary alignments.

Supplementary Data 5. Classification of *TCAF* gene models and isoforms across the eight haplogroups using FLNC transcripts. FLNC reads from one chimpanzee (*Pan troglodytes*) and six human tissues were generated using the PacBio Iso-Seq technology and used for the discovery of gene models and isoforms in each haplogroup. Only transcripts that can be mapped with high confidence (>99% sequence identity and more than 10 reads) and have an open reading frame with more than 200 amino acid (aa) were reported. Bolded isoform IDs are haplogroup-specific.

Supplementary Data 6. Coordinates of *TCAF* SDs on the long-read assembled BAC haplogroups.

Supplementary Data 7. Interlocus gene conversion (IGC) segments identified between *TCAF* SDs from BAC-assembled haplotypes using GENECONV (v.1.81a).

Supplementary Data 8. Geographic coordinates of the HGDP populations and summary statistics for the overall *TCAF* SD copy number (CN) estimates.

Supplementary Data 9. Summary of additional models used for coalescent simulations. In addition to models used in our original submission (Hsieh et al. 2019), we also generated simulations using demographic models implemented by the PopSim consortium—an open-source population genetics project (Stdpopsim; Adrion et al. 2020)— as well as models recently published for Siberians (Hsieh et al. 2017) and South Asians (Terhorst et al. 2019).

Supplementary Data 10. Numbers of windows in the *TCAF* locus in the extreme tails of genome-wide distribution of observed and simulated Tajima's *D* and Fay and Wu's *H*. Data were directly extracted from Fig. 5c and Supplementary Figs. 34-35. A total of 29 windows in the *TCAF* locus were used in the comparisons. The numbers in parentheses are the thresholds used to determine if the values of the statistics for individual windows fall within the extreme tails.

Supplementary Data 11. IsoSeq probe design sequences.
