# Supplementary material for: Evidence for opposing selective forces operating on human-specific duplicated TCAF genes in Neanderthals and humans
Source: Nat Commun. 2021 Aug 25;12:5118. doi: 10.1038/s41467-021-25435-4 (PMC8387397; doi:10.1038/s41467-021-25435-4)
Supplement: Supplementary file 15 — Reporting Summary [file 41467_2021_25435_MOESM15_ESM.pdf]

nature research

Corresponding author(s): Evan E. Eichler
PingHsun Hsieh

Last updated by author(s): Aug 2, 2021

Reporting Summary

Nature Research wishes to improve the reproducibility of the work that we publish. This form provides structure for consistency and transparency in reporting. For further information on Nature Research policies, see our Editorial Policies and the Editorial Policy Checklist.

Statistics

For all statistical analyses, confirm that the following items are present in the figure legend, table legend, main text, or Methods section.

n/a	Confirmed	
☐	☒	The exact sample size (*n*) for each experimental group/condition, given as a discrete number and unit of measurement
☐	☒	A statement on whether measurements were taken from distinct samples or whether the same sample was measured repeatedly
☐	☒	The statistical test(s) used AND whether they are one- or two-sided<br>*Only common tests should be described solely by name; describe more complex techniques in the Methods section.*
☐	☒	A description of all covariates tested
☐	☒	A description of any assumptions or corrections, such as tests of normality and adjustment for multiple comparisons
☐	☒	A full description of the statistical parameters including central tendency (e.g. means) or other basic estimates (e.g. regression coefficient) AND variation (e.g. standard deviation) or associated estimates of uncertainty (e.g. confidence intervals)
☐	☒	For null hypothesis testing, the test statistic (e.g. *F*, *t*, *r*) with confidence intervals, effect sizes, degrees of freedom and *P* value noted<br>*Give P values as exact values whenever suitable.*
☐	☒	For Bayesian analysis, information on the choice of priors and Markov chain Monte Carlo settings
☐	☒	For hierarchical and complex designs, identification of the appropriate level for tests and full reporting of outcomes
☐	☒	Estimates of effect sizes (e.g. Cohen's *d*, Pearson's *r*), indicating how they were calculated

Our web collection on statistics for biologists contains articles on many of the points above.

Software and code

Policy information about availability of computer code

Data collection

No code is used for data collection.

Data analysis

Analysis tools used in this study are listed as follows:
- mrsFAST (v3.4.1)
- Parasight (v7.6)
- Canu (v1.5)
- Arrow (v2.3.3)
- BLAST (v2.10.1)
- MAFFT (v7.453)
- GENECONV (v1.81a)
- Iso-Seq3 pipeline (v3.1.2)
- ANGEL (v3.0)
- minimap2 (v2.17-r941)
- BWA-MEM (v0.7.12)
- BWA (v0.7.17)
- IQ-TREE, (v1.6.11)
- BEAST (v2.5.0)
- ModelGenerator (v0.85)
- Tracer (v.1.7.1)
- Figtree (v1.4.3)
- BEAGLE (v5.1-25Nov19.28d)
- Rtsne (v0.15)
- rgdal (v1.5)

- scatterpie (v0.1.6)
- MaCS (v0.4e)
- PLINK (v1.09)
- FreeBayes (v1.0.2-6-g3ce827d)
- MEGA X (v10.1.8)
- msprime (v0.7.4)
- stdpopsim (v0.1.2)
- irlba (v2.3.3)

For manuscripts utilizing custom algorithms or software that are central to the research but not yet described in published literature, software must be made available to editors and reviewers. We strongly encourage code deposition in a community repository (e.g. GitHub). See the Nature Research guidelines for submitting code & software for further information.

Data

Policy information about availability of data

All manuscripts must include a data availability statement. This statement should provide the following information, where applicable:

- Accession codes, unique identifiers, or web links for publicly available datasets
- A list of figures that have associated raw data
- A description of any restrictions on data availability

All data used in this study have been uploaded and publicly available for download.
- Assembled bacterial artificial chromosome contigs (GenBank accession: MT985491 - MT985505 at https://www.ncbi.nlm.nih.gov/nuccore/)
- Iso-Seq capture transcript data (BioProject: PRJNA657884; https://www.ncbi.nlm.nih.gov/bioproject/?term=prjna657884)

In addition, publicly available data were downloaded from the source websites as described in the manuscript.
- Human Genome Diversity Project (https://www.internationalgenome.org/data-portal/data-collection/hgdp)
- Simons Genome Diversity Project (https://docs.cancergenomicscloud.org/v1.0/docs/simons-genome-diversity-project-sgdp-dataset)
- Neanderthal/Denisovan genomes (http://cdna.eva.mpg.de/neandertal/)
- Great ape nonhuman primate genomes (https://www.ncbi.nlm.nih.gov/bioproject/?term=PRJNA189439)
- GTEx multi-tissue data (release v8; https://gtexportal.org/home/)
- Human Protein Atlas (HPA; https://www.proteinatlas.org)

Field-specific reporting

Please select the one below that is the best fit for your research. If you are not sure, read the appropriate sections before making your selection.

☐ Life sciences ☐ Behavioural & social sciences ☒ Ecological, evolutionary & environmental sciences

For a reference copy of the document with all sections, see nature.com/documents/nr-reporting-summary-flat.pdf

Ecological, evolutionary & environmental sciences study design

All studies must disclose on these points even when the disclosure is negative.

Study description	The goal of this study is to reconstruct the evolutionary history of the thermo-regulatory gene family, TCAF, in modern and archaic humans using both long-read and short-read sequencing data. Short-read genomes were collected from several publicly available data set as described in the manuscript. Using long-read sequencing data, we characterized the complex human-specific duplications at this locus in multiple samples and used population genetics modeling, we are able to show opposing selective forces operating at this locus in Neanderthals and modern human populations. Our results also suggest differential TCAF expression among different duplication haplotypes in thyroid and peripheral nerve systems, implying their roles in possible cold and/or diet adaptations.
Research sample	- We leveraged a large collection of publicly available high-coverage Illumina genomes from the following sources. This collection collectively represents 1,102 contemporary human samples from a diverse panel of more than 127 populations across the world, 71 samples of four nonhuman great ape species (chimpanzee, bonobo, gorilla, and orangutan), three Neanderthal and one Denisovan individuals.<br><br>1) Human Genome Diversity Project (https://www.internationalgenome.org/data-portal/data-collection/hgdp).<br>2) Simons Genome Diversity Project (https://www.simonsfoundation.org/simons-genome-diversity-project/)<br>3) Great Ape Project (https://www.ncbi.nlm.nih.gov/bioproject/?term=PRJNA189439)<br>4) Neanderthal/Denisovan: (http://cdna.eva.mpg.de)<br><br>- For bacterial artificial chromosome sequencing, we used cell lines from the following sources. Samples were selected for sequencing to maximize the observed structural diversity at the TCAF locus.<br>1) Human BAC libraries: Virginia Mason Research Center.<br>2) Nonhuman primate libraries: Children's Hospital Oakland Research Institute.<br><br>- For full-length transcript data, the following sources for total RNA were used:<br>1) Dorsal root ganglion: Clontech Laboratories, Inc., Takara [catalog #636150]<br>2) Esophagus: Clontech Laboratories, Inc., Takara [catalog #636178]<br>3) Fibroblast (Coriell)<br>4) Skin: Biochain Institute Inc. [catalog #R1234218-P],

5) Fetal brain: NCBI BioSample: SAMN09459150
6) Testis: Clontech Laboratories, Inc., Takara [catalog #636533])

Sampling strategy	Our analysis focused on the evolution of a human-specific duplication locus, and thus the goal is to maximize the overall diversity in the data set rather than the number of samples. No sample size calculation was performed, and we depended on and collected publicly available sequencing data that can represent the global diversity in humans.
Data collection	Data were downloaded by PingHsun Hsieh, the first author of this study, to the local computer cluster at the University of Washington through Ethernet connections from the source websites as described in the Research Sample section above.
Timing and spatial scale	The study focus on the evolution of the TCAF locus in humans using both contemporary human (present day) and archaic hominin (>36,000 years) samples, which represent a sample from a diverse geographic locations across the world as shown in Fig. 1c in the main text.
Data exclusions	To ensure the genotype quality of single-nucleotide variant calling for unique diploid sequences at the TCAF locus (Methods), we excluded variants that were found within 10 bp of indels and have quality score (QUAL) < 20. We excluded 68 short-read samples due to having >10% missing data.
Reproducibility	We provide a list of all software packages used in this study as described in the Software and code section above. In addition, all corresponding parameters used for these software packages are documented in detail in the Methods section in the main text so the study can be reproduced accordingly.
Randomization	Samples were allocated into groups based on their population and/or ethnic origins as described in the publicly available source websites listed in the above Data section. Randomization procedures were used to assess statistical significance. In particular, to assess the significance of the observation of eight archaic hominin haplotypes that are virtually identical belonging to a single homogeneous group, we performed a test of 100 million permutations by randomizing the population labels among haplotypes and computing the frequency of such an observation.
Blinding	The evolutionary inferences were made using genetic variation data from samples collected from publicly available data sets. Thus, blinding is not possible in this study because it is based on already established and published datasets.

Did the study involve field work? ☐ Yes ☒ No

Reporting for specific materials, systems and methods

We require information from authors about some types of materials, experimental systems and methods used in many studies. Here, indicate whether each material, system or method listed is relevant to your study. If you are not sure if a list item applies to your research, read the appropriate section before selecting a response.

Materials & experimental systems

n/a	Involved in the study	
☒	☐	Antibodies
☐	☒	Eukaryotic cell lines
☒	☐	Palaeontology and archaeology
☒	☐	Animals and other organisms
☐	☒	Human research participants
☒	☐	Clinical data
☒	☐	Dual use research of concern

Methods

n/a	Involved in the study	
☒	☐	ChIP-seq
☒	☐	Flow cytometry
☒	☐	MRI-based neuroimaging

Eukaryotic cell lines

Policy information about cell lines

Cell line source(s)	1) Human BAC libraries: Virginia Mason Research Center.<br>2) Nonhuman primate libraries: Children’s Hospital Oakland Research Institute.
Authentication	Coriell institute performs cell line quality control procedures for authentication, including, but not limited to, karyotyping by G-banded analysis, FISH, Southern blot hybridization with minisatellite probes or by PCR using a panel of microsatellite markers, etc. Details about cell line quality control procedures can be found at Coriell Institute (https://catalog.coriell.org/0/Sections/Support/Global/QCcells.aspx?PgId=409)
Mycoplasma contamination	Cell lines were tested negative for mycoplasma contamination by the source institutions listed above.
Commonly misidentified lines (See ICLAC register)	No commonly misidentified cell lines were used in the study.

Human research participants

Policy information about studies involving human research participants

Population characteristics	Briefly,<br>1) Human Genome Diversity Project includes 54 global human populations (European Nucleotide Archive under study accession no. PRJEB6463).<br>2) Simons Genome Diversity Project includes ~100 diverse populations (https://www.simonsfoundation.org/simons-genome-diversity-project/)<br>3) Great Ape Project includes 79 samples from chimpanzee, bonobo, gorilla, and orangutan (http://www.ncbi.nlm.nih.gov/bioproject/189439)
Recruitment	All data were collected from publicly available data sets. No Recruitment was made.
Ethics oversight	All data were collected from publicly available data sets. Sample consents were made in those individual study.

Note that full information on the approval of the study protocol must also be provided in the manuscript.
